# A three-dimensional musculoskeletal model of the dog

**DOI:** 10.1038/s41598-021-90058-0

**Published:** 2021-05-31

**Authors:** Heiko Stark, Martin S. Fischer, Alexander Hunt, Fletcher Young, Roger Quinn, Emanuel Andrada

**Affiliations:** 1 Institute of Zoology and Evolutionary Research with Phyletic Museum, Friedrich-Schiller-University Jena, Jena, Germany; 2 Department of Mechanical and Material Engineering, Portland State University, Portland, USA; 3 Department of Mechanical and Aerospace Engineering, Case Western Reserve University, Cleveland, USA

**Keywords:** Biological techniques, Biological models, Musculoskeletal models

## Abstract

The domestic dog is interesting to investigate because of the wide range of body size, body mass, and physique in the many breeds. In the last several years, the number of clinical and biomechanical studies on dog locomotion has increased. However, the relationship between body structure and joint load during locomotion, as well as between joint load and degenerative diseases of the locomotor system (e.g. dysplasia), are not sufficiently understood. Collecting this data through in vivo measurements/records of joint forces and loads on deep/small muscles is complex, invasive, and sometimes unethical. The use of detailed musculoskeletal models may help fill the knowledge gap. We describe here the methods we used to create a detailed musculoskeletal model with 84 degrees of freedom and 134 muscles. Our model has three key-features: three-dimensionality, scalability, and modularity. We tested the validity of the model by identifying forelimb muscle synergies of a walking Beagle. We used inverse dynamics and static optimization to estimate muscle activations based on experimental data. We identified three muscle synergy groups by using hierarchical clustering. The activation patterns predicted from the model exhibit good agreement with experimental data for most of the forelimb muscles. We expect that our model will speed up the analysis of how body size, physique, agility, and disease influence neuronal control and joint loading in dog locomotion.

## Introduction

The Dog (*canis lupus* f. familiaris) is interesting to investigate because of the wide ranges of body size, body mass, and physique of their more than 400 globally recognized breeds^1^.

There exists an important body of work related to kinematic and dynamical differences between healthy dogs and dogs with musculoskeletal diseases^2–5^. However, the relationship between body structure and joint load during locomotion, as well as between joint load and degenerative diseases of the locomotor system (e.g. dysplasia), are not sufficiently understood. To investigate how body size, physique, agility, and diseases influence joint control and load in dogs, it is necessary to model the morphology﻿﻿ with the external and internal forces that produce locomotion. To analyze joint mechanics, inverse dynamic analysis is typically used^6–9^. Inverse dynamics analysis is a method of the engineering sciences that combines kinetic, kinematic, and morphometric data to provide an indirect way to describe the causes of movement patterns. In order to quantify the joint load, the internal transmission of force through the skeleton, and consequently the generation of force in the muscles is required^10^. Simulated models, rather than invasive methods, are best suited to evaluate force transmission between segmental elements^10–15^. Specific dog musculoskeletal models exist for the hindlimbs^11,16–18^. However, a model with all four legs and the musculoskeletal area between them is necessary to address questions about adaptivity. For example, in the case that one limb is injured or perturbed, a whole model can help to understand compensation mechanisms at joint and muscular levels in every limb. In addition, it could help to analyze how reflexes, central pattern generators, and higher locomotion centers control those adaptations.

Whenever a model is developed to test a scientific hypothesis, the amount of complexity required to address the question must be determined^19^. Investigations of the general behaviour of the whole system (global dynamics) require a different approach than the analysis of joint mechanics or joint load. Thus, one needs to choose between simple models such as the spring-mass-model^20^, more complex multi-body models, or detailed models of body parts using the Finite Element Method (FEM). Model parameters (constant quantities during the simulation, e.g. mass or geometry), and model variables (speed, forces), must be obtained from experiments, literature, or ‘educated guesses’. Thus, the availability of model parameters and variables can influence the model’s complexity. In general, simple models (also termed templates by Full and Koditschek^21^) are well suited to study the basic principles of movement, while more complex models (termed anchors by Full and Koditschek^21^) provide more detailed insights. A mixture of both extremes can be used to break-down the multidimensionality of complex models^22^.

**Figure 1:**
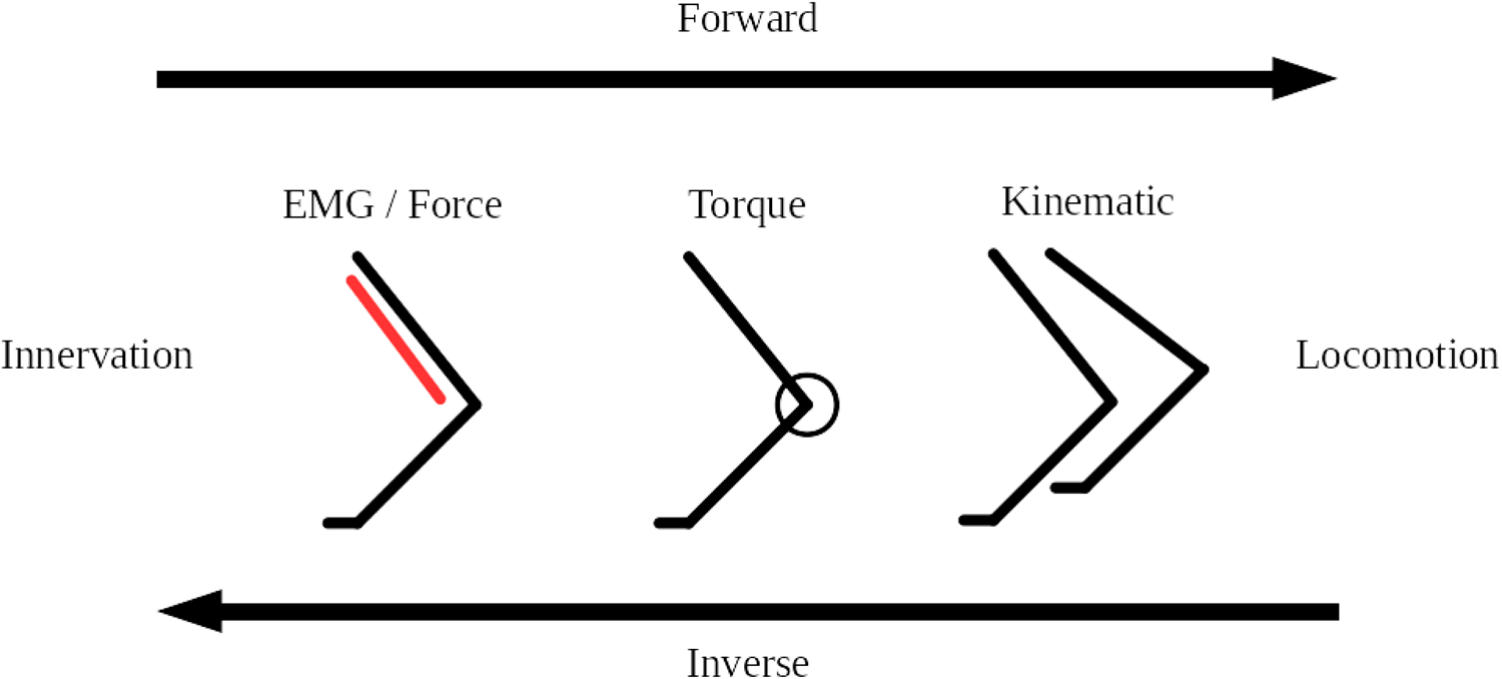
Abstraction of the forward and inverse simulation parameter chain for forelimb locomotion. Depending on the direction of the examination, the chain starts on the left or right side. The figure was created with the software package LibreOffice^71^.

Two distinct methods are used to generate simulations (Fig. 1). Forward simulations calculate specific torques in the joints on the basis of innervation data, electromyography (EMG) data, or muscle forces. These forces generate joint movement and finally locomotion through interactions with the environment. An inverse simulation, in which the joint torques are calculated from kinematic and kinetic data, is used to find muscle forces, muscle activation patterns, or innervation data *a posteriori* via static or dynamic optimization. The transformation of torques into muscle forces is done by numerical approximation based on muscle insertion points and other anatomical data. Note that for any recorded joint kinematics and joint torques, the muscle forces solution is not unique (underdetermined system)﻿.

### Aim

We describe here the methods we used to create a detailed musculoskeletal model of a specific dog breed, the Beagle. Beagles are often used for experimental purposes and in veterinary education and are well suited for a generalized model. To permit a broad use of the model, we identified three key-features: three-dimensionality, scalability, and modularity. Three-dimensionality is needed to represent a variety of movements such as periodic and non-periodic locomotion, agility, or ideomotion (e.g. scratching). Scalability is important to include because the body and limb lengths vary even within a dog breed. Scalability also allows the model to be useful in assessing different breeds. Modularity helps to adapt the model to the requirements/limitations of the experimental setup (e.g. single leg or multi-leg analysis).

A second and specific goal of the present work is the identification of muscle synergies for a defined limb motion based on model simulations. This avoids invasive muscle activation recording and enables the inclusion of small and deep muscles. Muscles’ synergies are also important for the design and modelling of neuronal circuitry^23,24^. For determining muscle synergies, we used inverse dynamics and static optimization to estimate muscle activations based on experimental data of a walking Beagle. We present joint torques, muscle activations, and synergistic muscle groups of the forelimb. Torque profiles and muscle activation are then compared with published results.

## Results

### Dog model

The complete model has a maximum of 84 DOFs. Head, thorax, abdomen, and tail have three rotational DOFs each. Vertebral motions were not modelled. We included 134 (67 per side) muscles (with their corresponding muscle parameters) encompassing the majority of fore- and hindlimb muscles and the epaxial muscles relevant for locomotion. Head, belly, and toe muscles were not included. The complete set of muscles can be found in (Suppl. 5 STab. 2).

### Left forelimb

To evaluate the model, the left forelimb was chosen as the test case. For simplicity, most of the muscles were modelled as single lines. However, the muscles that wrap over the joint have more segments to match joint constraints. The geometric constraints are necessary to avoid bone penetration. As expected, the number of degrees of freedom per joint influenced the results. For the forelimbs, 15 DOFs were necessary for static optimization convergence: scapula (5 DOFs), shoulder (3 DOFs), elbow (2 DOFs), carpal joint (2 DOFs), paw (3 DOFs) (details see Table 1). For example, without scapular anterior-posterior translation, *M. rhomboideus* and *M. latissimus dorsi* activation peaked during the early stance phase. Such an activation profile has not been reported in the literature. However, when the translational DOFs in the scapular joint were allowed, the *M. rhomboideus* was activated continuously, and the *M. latissimus dorsi* was active at the end of the swing phase, matching experimental observations^25–27^. The increment of the DOFs to 16 (including mediolateral translation of the scapula) worsened simulation results (e.g. the activations of the muscles *M. trapezius* and *M. rhomboidues pars thoracica* were silent).

**Table 1:** The maximum force and torque actuator values of the joints of the left forelimb calculated with a parameter map. As well as the degrees of freedom in the joints.

	Global	Scapula	Shoulder	Elbow	Carpal joint	Forepaw
Mx (Nm)	–	5	0.0005	1	5	0.005
My (Nm)	–	5	5	5	–	0.005
Mz (Nm)	–	5	5	–	1	0.0005
Fx (N)	0.5	–	–	–	–	–
Fy (N)	0.5	0.00005	–	–	–	–
Fz (N)	0.5	0.5	–	–	–	–
DOFs	3	5	3	2	2	3

### Torques

**Figure 2:**
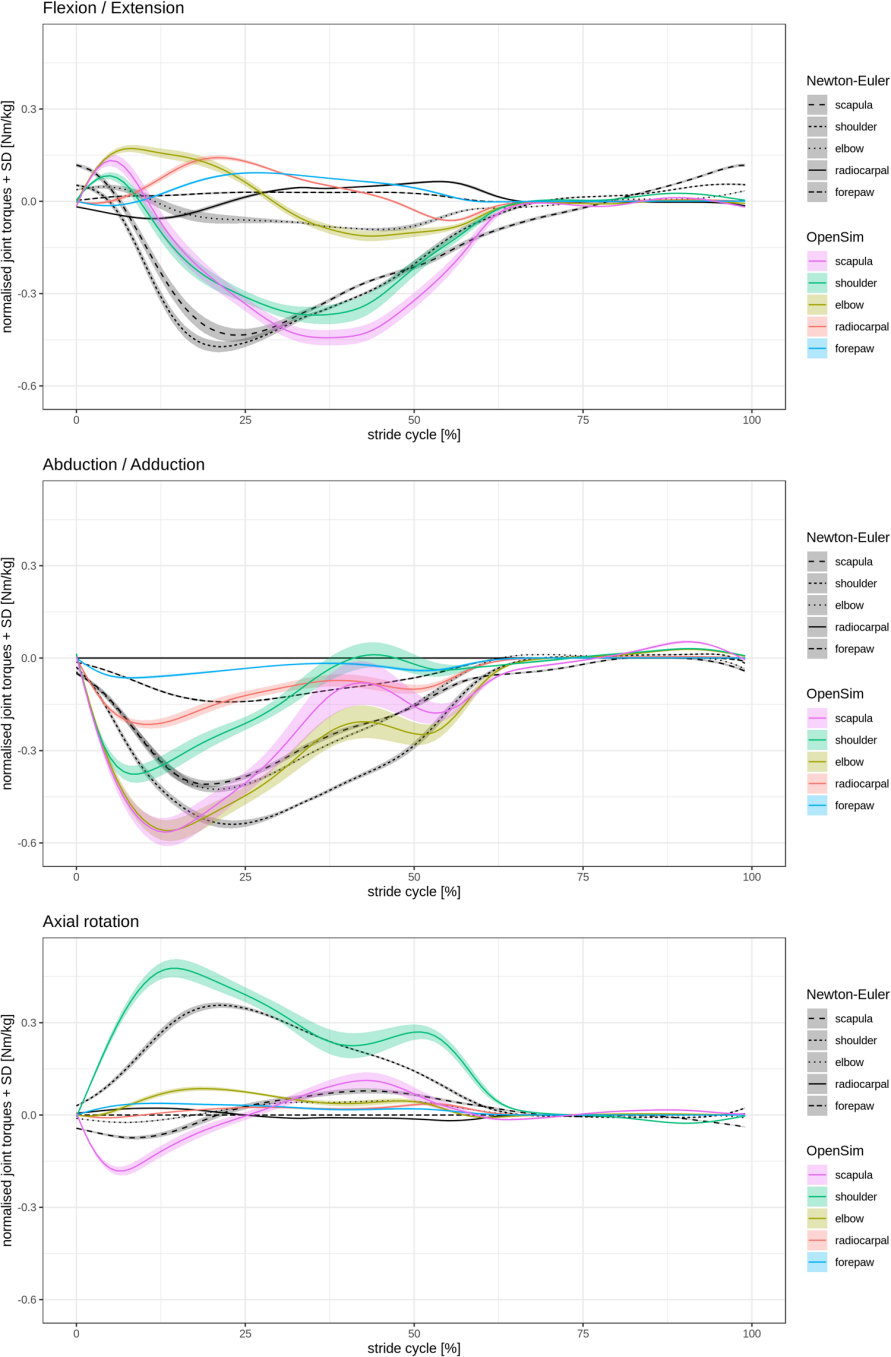
Forelimb calculated torques for the flexion/extension, abduction/adduction, and axial rotation in a walking Beagle based on consecutive strides of the same trial. Comparison between results obtained from OpenSim versus Newton–Euler method. The standard deviation (SD) is shown as shaded bands. Flexion/extension torques: positive values indicate net retractor torques and negative values indicate net protractor torques. A retractor torque flexes the shoulder joint, extends the elbow joint, and flexes the carpal joint. Abduction/adduction torques: negative values indicate abductor torque and positive values indicate adductor torque. Axial rotation torques: negative values indicate external rotation torques and positive values indicate internal rotation torques. The figures were created with the software package R^70^.

The comparison between torque results computed by OpenSim^28,29^ and those computed using the Newton–Euler method (see supplement for method details Suppl. 4) show more agreement in the flexion/extension (Fig. 2). The abduction/adduction displays higher discrepancies (Fig. 2). In the axial rotation, differences can be observed for the scapula and the humerus (Fig. 2). Still, the torque amplitudes and patterns computed using OpenSim^28,29^ are similar to those computed using the Newton–Euler method and to other published results^12,30^ (Fig. 2). The scapula and the humerus displayed the largest torque amplitudes in flexion/extension. Their torque displayed similar amplitudes, and pattern changes from positive to negative. In abduction/adduction, besides the carpal joint and the forepaw, all torque patterns and amplitudes were similar. In axial rotation, the shoulder joint displays larger torque amplitudes.

### Muscle activation

**Figure 3:**
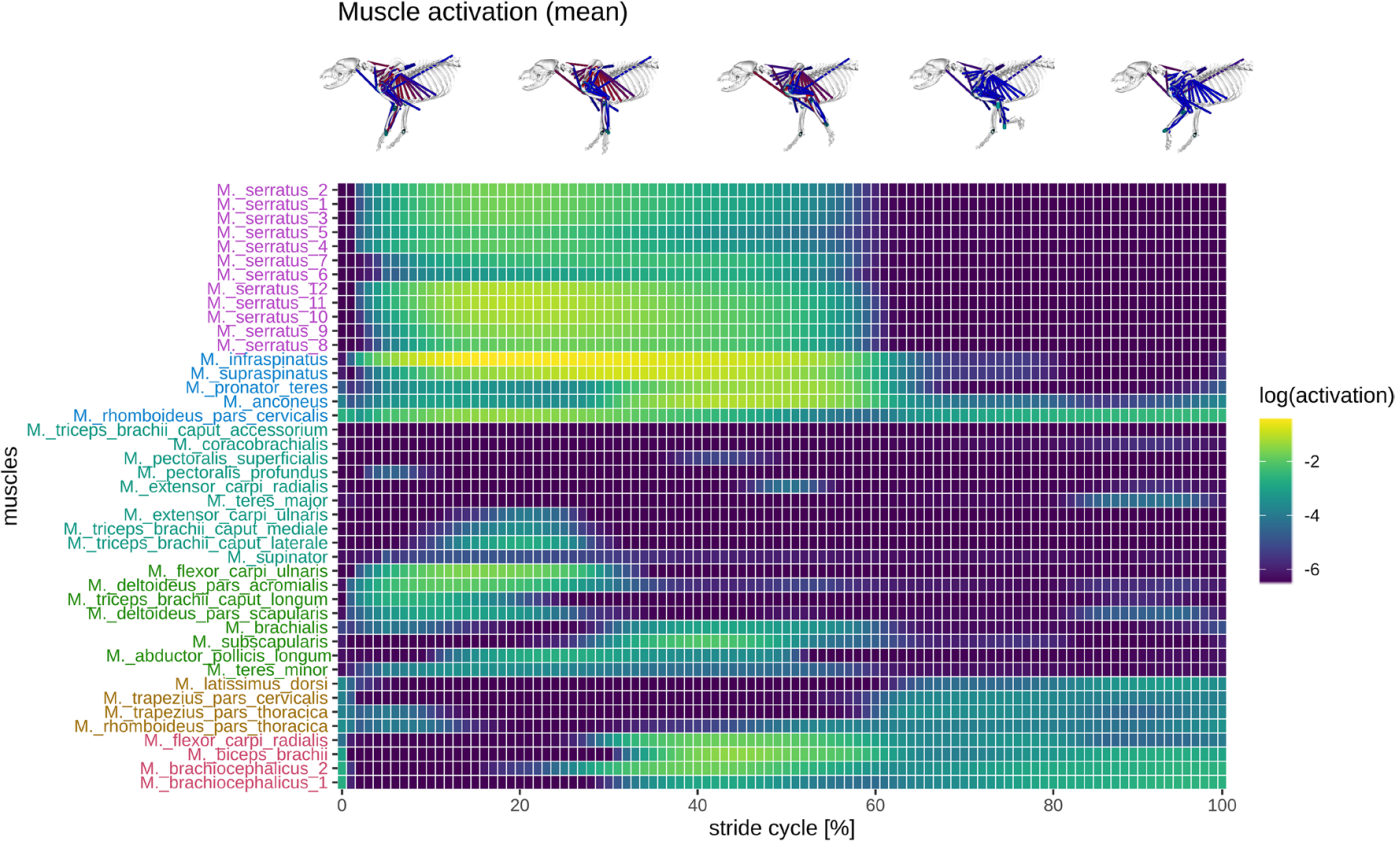
Simulated forelimb muscle activation in a walking Beagle during one gait cycle, shown as a heatmap of logarithmic values (log2). The plot shows how individual muscles were activated based on consecutive strides of the same trial. The muscle groups (colours) were arranged according to hierarchical clustering (method—ward.d2) and minimal leaf sorting. The figures were created with the software packages OpenSim^28,29^ and R^70^.

The muscle activations calculated through static optimization were similar to those collected in experiments^25,27^ (Suppl. 6 SFig. 10, 11, 12 and 13). The muscles that exhibited the largest activations were *M. supraspinatus* and *M. infraspinatus* (Fig. 3; Suppl. 6 SFig. 7#1). Both muscles were activated during the whole stance phase. All other muscles were only activated during either the early or late stance phase. The *M. serratus* demonstrated a cranial-to-caudal travelling activation wave around touch down and toe-off. Note that the cranial parts retract the leg, and the caudal parts protract the leg.

**Figure 4:**
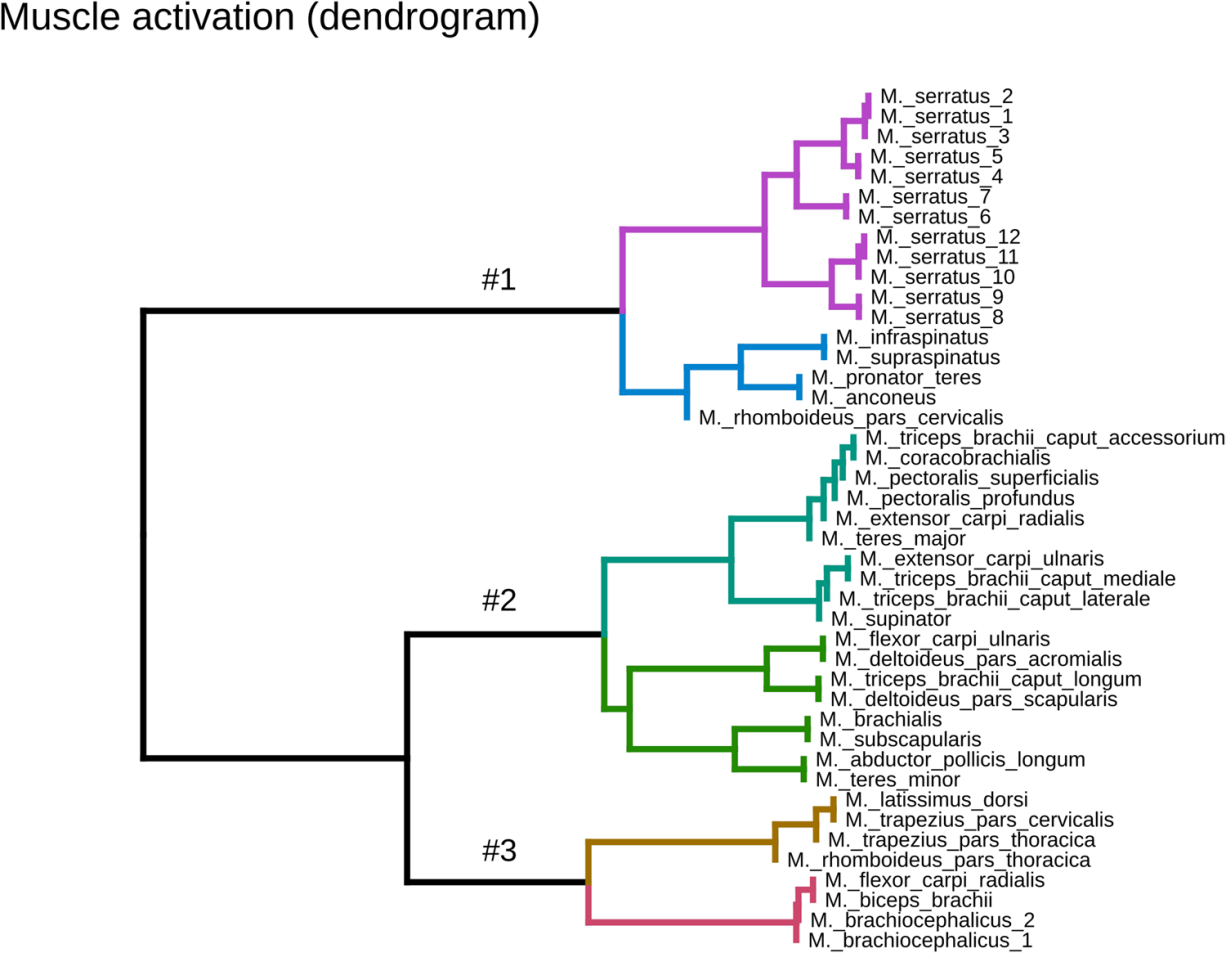
Hierarchical clustering (method—ward.d2) and minimal leaf sorting of simulated forelimb logarithmic muscle activation (log2) in the walking Beagle. The dendrogram shows how individual muscles were activated based on consecutive strides of the same trial. Groups represent a distance between activation patterns. The distance is displayed in the dendrogram as branch length. The longest lengths from the root were used as criteria to separate groups. The figure was created with the software package R^70^.

The hierarchical cluster-analysis separated muscles into three main synergistic groups (Fig. 4—black lines; Suppl. 6 SFig. 8 and 9). Every main synergistic group has two subgroups (see different colours in Fig. 4). The purple subgroup of group #1 includes all of the different parts of *M. serratus*. The blue subgroup of group #1 includes *M. infraspinatus*, *M. supraspinatus*, *M. pronator teres*, *M. anconeus*, and *M. rhomboideus pars cervicalis*.

The cyan subgroup of group #2 encompasses a large number of joint extensor and joint stabilizing muscles and includes two minor groups. The first minor group includes *M. triceps brachii accessorium*, *M. coracobrachialis*, both parts of *M. pectoralis*, *M. extensor carpi radialis*, and *M. teres major*. The second minor group of muscles within the cyan subgroup include: *M. extensor carpi ulnaris*, *M. supinator*, *M. triceps brachii caput mediale*, and *M. triceps brachii caput laterale*.

The green subgroup #2 includes *M. flexor carpi ulnaris*, *M. deltoideus pars acromialis*, *M. triceps brachii caput longum*, *M. deltoideus pars scapularis*, *M. brachialis*, *M. subscapularis*, *M. abductor pollicis longum*, and *M. teres minor*.

The brown subgroup of group #3 includes *M. latissimus dorsi*, *M. trapezius pars cervicalis*, *M. trapezius pars thoracica*, *M. rhomboideus pars thoracica*. The magenta subgroup of group #3 contains *M. flexor carpi radialis*, *M. biceps brachii*, and both parts of the *M. brachiocephalicus*.

## Discussion

Dogs have more than 400 globally recognized breeds^1^. Thus, they enable an interesting analysis of how body size, physique, and agility, as well as diseases, influence joint control in quadrupedal locomotion. The aim of this work was to develop a detailed, fully three-dimensional, and scalable musculoskeletal model of a dog to analyze these effects.

We designed a flexible framework that can be used to generate different dog models ranging from individual leg components up to an entire musculoskeletal model. Additionally, this framework can be used to generate models for different breeds. To our knowledge, our model is the first fully three-dimensional model of a dog, and contains 134 (67 per side) muscles including the majority of fore- and hindlimb muscles and epaxial muscles relevant for locomotion.

To evaluate the model, we calculated torques and muscle activation patterns for the left forelimb using inverse dynamics. We compared the results of the inverse dynamic tool in OpenSim^28,29^ to the inverse dynamic computations based on the same data using the Newton–Euler method and published data. The right forelimb is a mirrored copy of the left one, thus we are confident that results gathered from the left leg will also be applicable to the right leg. The hind limbs have all necessary elements (segments, joints, joint actuators, muscles, and their corresponding muscle parameters) to perform simulations, however, they still need to be validated.

Only a few available studies on 2D-inverse dynamic analysis of the canine forelimb exist (i.e. excluding shoulder joint and scapular fulcrum^4,5,12^) and only one three-dimensional analysis of the whole forelimb^30^. Note that only Nielsen et al.^12^ and Andrada et al.^30^ reported on healthy dogs, and therefore the results of Burton, et al.^4^ and Burton et al.^5^ will not be discussed here.

As a standard configuration, OpenSim^28,29^ presents joint-torques about joint axes. For comparison, we also computed joint-torques in the joint’s coordinate system. Local coordinates provide an effective method for checking the functionality and validity of the model, and can be easily transformed into anatomical ones. The torque amplitudes and patterns about the mediolateral axis computed with OpenSim^28,29^ were similar to those computed using the Newton-Euler method and to published results^12,30^ (Fig. 2). Differences in torque amplitudes were observed for the abduction/adduction and axial rotation. Discrepancies between OpenSim^28,29^ and Newton-Euler methods are to be expected because of the error accumulation in the recursive Newton-Euler method and differences in filtering. In addition, the position of the joints have more variability in marker data and length constraint is not guaranteed in our Newton-Euler computation. Moreover, we can not be sure how OpenSim^28,29^ models the Cardan-sequence of the relative joint angles. Even small differences in leg orientation related to the ground reaction force (GRF) vector might also help to explain the discrepancies observed, especially in the abduction/adduction. In general, multi body systems have less computational error^31^.

OpenSim^28,29^ uses a two-step process to estimate muscle activation: First, inverse dynamics are used to compute joint forces and then static optimization is used to compute muscle forces. OpenSim^28,29^ also offers a more advanced method named computed muscle control (CMC). This method combines inverse static optimization with forward dynamics. The aim of this work was not to accurately predict detailed muscle activations and draw conclusions on exact timing and magnitude, but to roughly validate the model and determine if muscle locations and parameters were in the correct range. Therefore, we used the inverse dynamics plus static optimization method as it provides faster results with fewer parameters.

For the simulations, we chose the Hill-type muscle model by Millard et al.^32^. We took muscle parameters from the works by Shahar and Milgram^33,34^ and Williams et al.^35,36^ and linearly scaled them to the body mass of a Beagle. Additionally, we estimated the tendon length (see methods). As a general rule, muscle contractions in OpenSim^28,29^ are not effective in stabilizing joints while also reproducing limb kinematics during walking. Therefore, we included additional actuators in all joints to assist the muscles. A parameter search found the minimal actuator force/torque values that permitted the static optimization to converge.

To help validate musculoskeletal models, the prediction of muscle activation is often compared to EMG data collected from the same individuals. We did not collect EMG data from the dog modeled in these simulations, and this is a limitation of this study. Instead, we used EMG available in the literature as means of comparison to our model. The reader should be aware that muscle data does not exist for all muscles, furthermore, for some muscles there is only one source. This is why we compare our simulation results sometimes with just one literature source.

Model predictions of muscle activations, including the correct separation of the muscle groups with regard to the stance and swing phase, showed good agreement with reported EMG data^25–27^ (Suppl. 6 SFigs. 10, 11, 12 and 13). Exceptions were *M. triceps* and *M. trapezius* (compared to Tokuriki^25^), *M. pectoralis* (compared to Tokuriki^25^ & Deban et al.^27^), and *M. serratus ventralis cervicalis* (compared to Deban et al.^27^). Muscle activation is sensitive to the assigned muscle characteristics, joint actuator parameters, and the muscle redundancy solver (static optimization). A sensitivity analysis could provide insight into what specific muscle/joint properties have the most effect on muscle activation patterns. However, such an analysis is outside the scope of the present work. Additionally, muscle activation patterns in the literature are sometimes inconsistent (*M. pectoralis profundus* $\Rightarrow$ Tokuriki $\ne$ Deban; *M. latissimus dorsi* $\Rightarrow$ Deban $\ne$ Tokuriki, Suppl. 6 SFig. 13D,E), making the comparison to a ground truth difficult. The position of the electrodes (especially in large muscles), time-varying activation of different muscle regions, and muscle cross-talk may explain these differences. This, however, cannot be tested, as Tokuriki^25^ did not report the position of the electrodes. Our findings did show that the translational anterior-posterior and vertical DOFs must be included in the scapular joint for a correct prediction of shoulder muscles. For example, predictions of the activation patterns of the *M. rhomboideus* and *M. latissimus dorsi* are similar to those reported in the literature only when the scapular joint has five or six DOFs. Anterior-posterior translation in the scapular joint must be present as observed in kinematic studies (see Fujiwara^37^ for review). The fact that the scapula is only linked to the body with muscles indicates that the scapular joint might hold an additional role as a damper, reducing the propagation of impact forces to the body (e.g. after jumping) and minimizing the necessity of gait compensation mechanisms.

We used a hierarchical cluster-analysis to further analyze the validity of our musculoskeletal model. This analysis identifies synergistic groups by organizing muscles based on their activation patterns. This method is widely used in the literature to classify motor neuron activation, movement, or disease-related differences^38–40^. We used this method, in addition to the information about synergies, to test how well our simulations match literature data and to compare how well different data sources match each other.

The theory of muscle synergies^41–43^ hypothesizes that the central nervous system (CNS) produces different motor behaviours by co-activating groups of muscles in space or time^41,44^. Two types of muscle synergies have been identified^44,45^: (1) ‘synchronous synergies’, which activate a group of muscles at the same time; and (2) ‘time-varying synergies’, which produce patterns with a temporal profile for each muscle of a synergistic group. Muscle synergies is a theory for understanding how the CNS produces a wide range of motor behaviours and could be an important tool to simplify the control problem in complex neuromechanical models^24,46^.

Synergy decomposition yielded three main muscle groups within our model. Each of these groups was further divided into two subgroups, which are denoted by different colours in Fig. 4 (group #1: purple and blue, group #2: cyan and green, group #3: brown and magenta). Branches within one colour indicate small differences in the activation profiles. The purple subgroup of group #1 belongs to all the different parts of the *M. serratus*. Those parts were activated sequentially from the most cranial to the most caudal parts. The cranial parts protract, while the most caudal parts retract the scapula. The protraction of the scapula correlates with the braking GRFs observed in most of the stance phase, while the retraction correlates with the acceleration phase in late stance.

The blue subgroup of group #1 includes *M. infraspinatus*, *M. supraspinatus*, *M. pronator teres*, *M. anconeus*, and *M. rhomboideus pars cervicalis*. *M. supraspinatus* and *M. infraspinatus* extend the shoulder joint, *M. anconeus* extends the elbow joint, while *M. rhomboideus pars cervicalis* mainly stabilizes the scapular joint. In our simulations, they were recruited during stance and part of the swing phases. However, only *M. rhomboideus pars cervicalis* was active throughout the entire stride. EMG data exists for *M. supraspinatus*, *M. infraspinatus*, and *M. rhomboideus pars cervicalis*. Our simulation results display a good agreement with those experimental data^25,27,47^.

The cyan subgroup of group #2 encompasses a large number of muscles. Among this subgroup, two main activation patterns were predicted. The first group encompasses the *M. triceps brachii accessorium*, *M. coracobrachialis*, both parts of *M. pectoralis*, *M. extensor carpi radialis*, and *M. teres major*. Those muscles showed minimal activations during simulations, which differs from published EMG data. One explanation for these differences is that more accurate muscle parameters and/or﻿﻿ new goal functions for optimization are needed to better distribute force among muscles. A further explanation could be, based on the fact that these muscles are difficult to measure, that the published data displayed just a cross-talk to more superficial muscles. We speculate that these muscles may be recruited for other tasks (e.g. perturbed locomotion).

The second group of muscles among the cyan subgroup (*M. extensor carpi ulnaris*, *M. triceps brachii caput mediale*, *M. triceps brachii caput laterale*, and *M. supinator*) were recruited in the early stance phase. They work mainly against gravity and control the axial function of the leg. The axial function refers to the time-dependent length and applied GRFs of the leg as measured from the main proximal pivot/fulcrum (scapular “joint”, that can be better described as an instantaneous centre of rotation) to the foot^48–50^.

The green subgroup of group #2 is made up of the following muscles: *M. flexor carpi ulnaris*, *M. deltoideus pars acromialis*, *M. deltoideus pars scapularis*, *M. triceps brachii caput longum*, *M. brachialis*, *M. subscapularis*, *M. abductor pollicis longus*, and *M. teres minor*. In the literature, there exists EMG data only for *M. brachialis*. Experimental data show that during walking, this elbow flexor is recruited from late stance until approximately mid-swing^47^. In our model, the *M. brachialis*, while displaying similar whole activation time, started about 10% of the stride time earlier than in the experiments. Interestingly, in our simulations, *M. subscapularis* had a similar activation pattern to those predicted for *M. brachialis*, *M. abductor pollicis longus*, and *M. teres minor* were activated earlier in the stance phase. The former was activated around mid-stance, while the latter was activated during the early stance phase. *M. teres minor* displayed a similar activation pattern to the EMG data published for *M. teres major*^47^. This could indicate that *M. teres minor* took the place of the *M. teres major* in our simulations. However, turning-off *M. teres minor* in simulations did not significantly improve the predictions of the activations of *M. teres major*.

The third group encompasses muscles that were activated throughout the swing phase. Some of them were also recruited during parts of the stance phase. The brown subgroup of group #3 includes *M. latissimus dorsi*, *M. trapezius pars cervicalis*, *M. trapezius pars thoracica*, and *M. rhomboideus pars thoracica*. The predicted activation of *M. latissimus dorsi* resembles experimental findings. It brakes the protraction of the forelimb during swing before touchdown. On the other hand, *M. trapezius* and *M. rhomboideus* stabilize the scapular joint. In experiments, *M. trapezius pars cervicalis* was active during the entire stride cycle, while *M. trapezius pars thoracica* was active during the complete stance phase and at the late swing phase^47^. Our simulations predicted *M. trapezius* to be active earlier during stance and during the complete swing phases. EMG data shows that *M. rhomboideus pars thoracica* is active during most of the stride cycle with the exception of a short period around mid-swing. In our simulations, this silent period occurs around mid-stance.

The magenta subgroup of group #3 contains *M. flexor carpi radialis*, *M. biceps brachii*, and both parts of *M. brachiocephalicus*. The first two muscles flex the paw and elbow joint, respectively, while the third protracts the forelimb. In the literature, it was shown that these muscles have similar activation patterns. They are briefly active after touchdown, then around take-off, and in the late swing phase. Our simulations display a similar pattern to those found in experiments. Furthermore, they show that the largest activations occur around take-off in preparation and start of the swing phase.

## Conclusions

We have developed a musculoskeletal model of a dog that has three main features: three-dimensionality, scalability, and modularity. Activation patterns predicted by static optimization exhibited good agreement with experimental data for most of the forelimb muscles. However, because muscles were unable to stabilize joints on their own, joint actuators have been included for stability. In animals, joints are stabilized by muscle co-contraction and passive structures^51–54^. Thus, muscle geometry, muscle parameters, and the modelling of passive structures are essential for an accurate estimation of muscle activation. To this end, more detailed breed-related anatomical and physiological studies are necessary. Other optimization algorithms such as computed muscle control (CMC) from OpenSim^28,29^ or predictive forward simulations might also improve the predictive power of this dog model^55^. We expect that the use of our model will speed up the analysis of how body size, physique, and agility (as well as diseases) influence joint control and loading in dog locomotion. We follow two different paths for the expansion of this model: (a) we are modelling specific joints in more detail using the finite element method to analyze joint loads based on the force data of the current model; (b) we intend to expand the model to a neuromechanical model^23,24^, to understand how the neural, muscular and skeletal systems operate together to produce efficient and stable locomotion. To this end, we also presented a method to estimate muscle synergies, which can help to break-down the design complexity of neuronal networks.

## Methods

In the present study, only existing animal data^30^ and literature data^33–36,47,56^ were used. In particular, the motion analysis performed by Andrada et al.^30^ was approved by the German Animal Welfare of the states of Thuringia and Lower Saxony (Registration No. TLV Az. 22-2684-04-02-012/14, LAVES 33.9-42502-04-14/1518), and carried out in strict accordance with their guidelines.

### Computed tomography data

**Figure 5:**
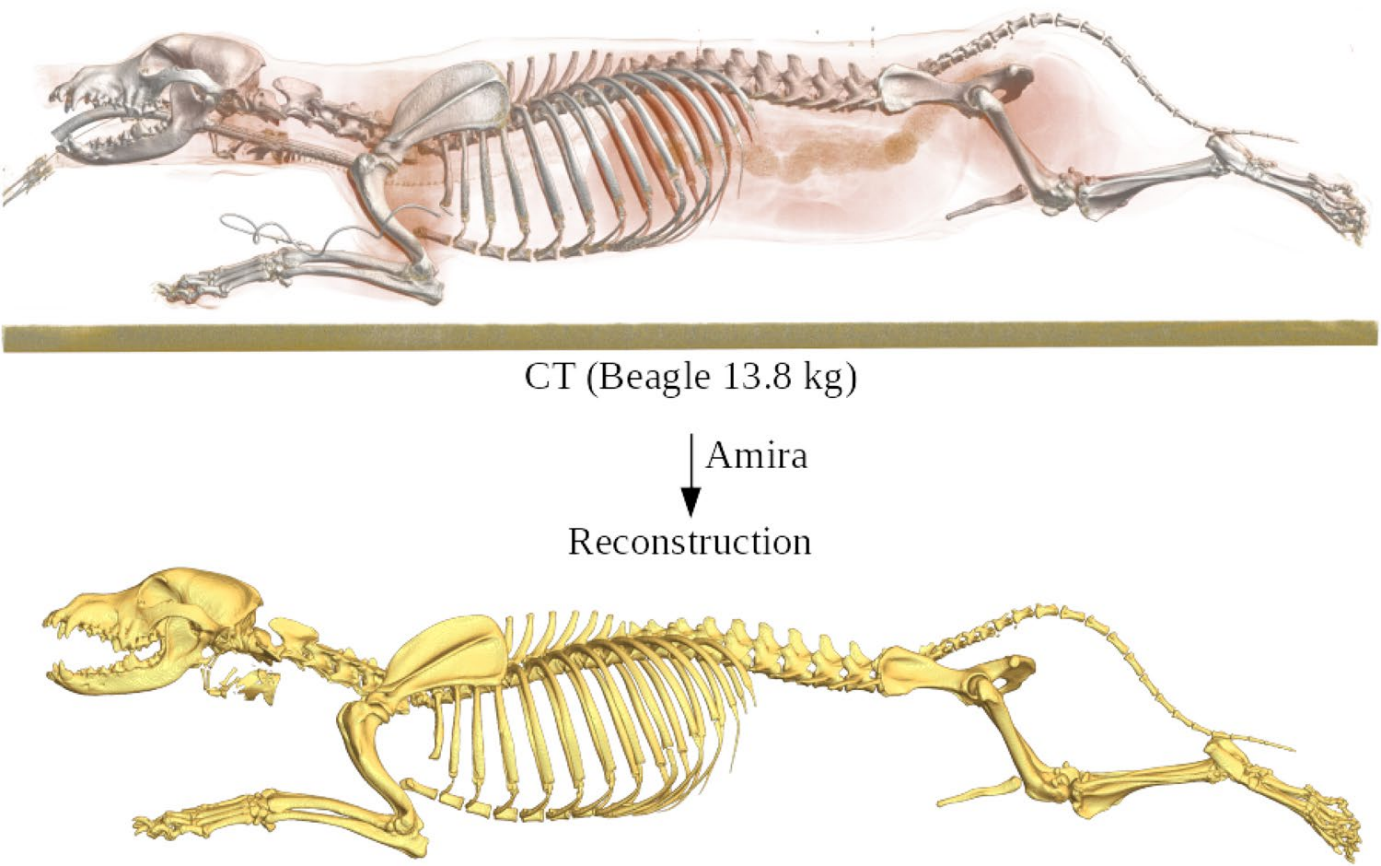
High-resolution computed tomography (CT) data set of an anaesthetized Beagle (upper image—lateral view) and the reconstruction of the separated bones (lower image—lateral view) using the software package Amira^57^.

The Beagle model (BE-model) was built using computed tomography (CT) data of an adult Beagle (13.8 kg; Andrada et al.^30^). The resulting CT data set consisted of 3370 (spacing 0.33 mm) sections with a resolution of $512 \times 512$ pixels (spacing 0.$279 \times 0$.279 mm^2^). All skeletal bones and muscle attachment points were reconstructed from the CT data (Fig. 5)

.

For reconstruction, we use the segmentation software Amira^57^ and the analysis software imageXd^58^ for automatic mesh generation. In addition, the segment masses^30,56^ and their moments of inertia were determined from the CT data (see Suppl. 2 and 3).

### Muscle data

To initialize the placement of muscles on the skeletal model, we used an existing detailed anatomical model of the working line of the German Shepherd dog (GS-model) by J. Laustrëer, A. Andikfar & M.S. Fischer^47^ and transformed it to the Beagle using the Beagle’s skeletal model and insertion points. The GS model is based on cross-sections of the limbs and body as well as macroscopic dissections. The GS-model was created originally for illustration and animation in Autodesk Maya^59^, and cannot be directly used in simulation tasks. In that model, muscle paths were modelled as nurbs (non-uniform rational basis splines), which were spatially aligned transversely to the fibre course. The reconstruction of the muscle centrelines from the nurbs was performed in ‘Cloud2’ software^58^ (Suppl. 5 and 6 SFig. 1).

The muscle parameters were taken from Shahar and Milgram^33,34^ and Williams et al.^35,36^. Shahar & Milgram published morphometric data of one hindlimb and four forelimbs of mixed-breed dogs. The morphometric variables included the muscle mass (*m*), muscle length (*ml*), muscle fibre length (*fl*), angle of pennation ($\alpha$), and the resulting physiological cross-sectional area (*PCSA*). In addition, Williams et al.^35,36^ published morphometric data of seven forelimbs and six hindlimbs of racing Greyhounds. Here the morphometric variables included muscle mass (*m*), muscle length (*ml*), fascicle length (*fl*), as well as PCSA, maximum force, and power. The tendon length (*tl*), which is important for the model (Suppl. 6 SFig. 2), was not available and thus approximated using the following formula:

$$\begin{aligned} tl = ml - \left( fl * cos \left( \alpha \right) \right) \end{aligned}$$

Adaptation or scaling of muscle parameters from other breeds or species is always a compromise. For the GS-model we used the muscle parameters from Shahar and Milgram^33,34^ and Williams et al.^35,36^. To scale those parameters for the BE-model, we tested whether mass and total leg muscle PCSA scales with body mass for the published data. We found logarithmic relationships between both mass and total PCSA for a limb and body weight. Those relationships were used to scale every muscle PCSA to our BE-model (Suppl. 6 SFig. 3 and 4). Parameters that scale with length (e.g. muscle fibre, tendon slack length) are automatically scaled with the geometric change in OpenSim^28,29^.

### Model assembly

To permit higher flexibility and broader use of the model, we generated the model in a way that it can be compiled in different scripting languages. The basic script was written in Master^58^, compiled as SIMM language^60^, and then converted via the simmToOpenSim tool^28,29^ into the OpenSim language^28,29^. In addition, the scripts were created in such a way that we can flexibly create models with different specifications. Depending on the necessity, we can create the whole dog model or parts of it such as fore- or hindlimbs.

**Figure 6:**
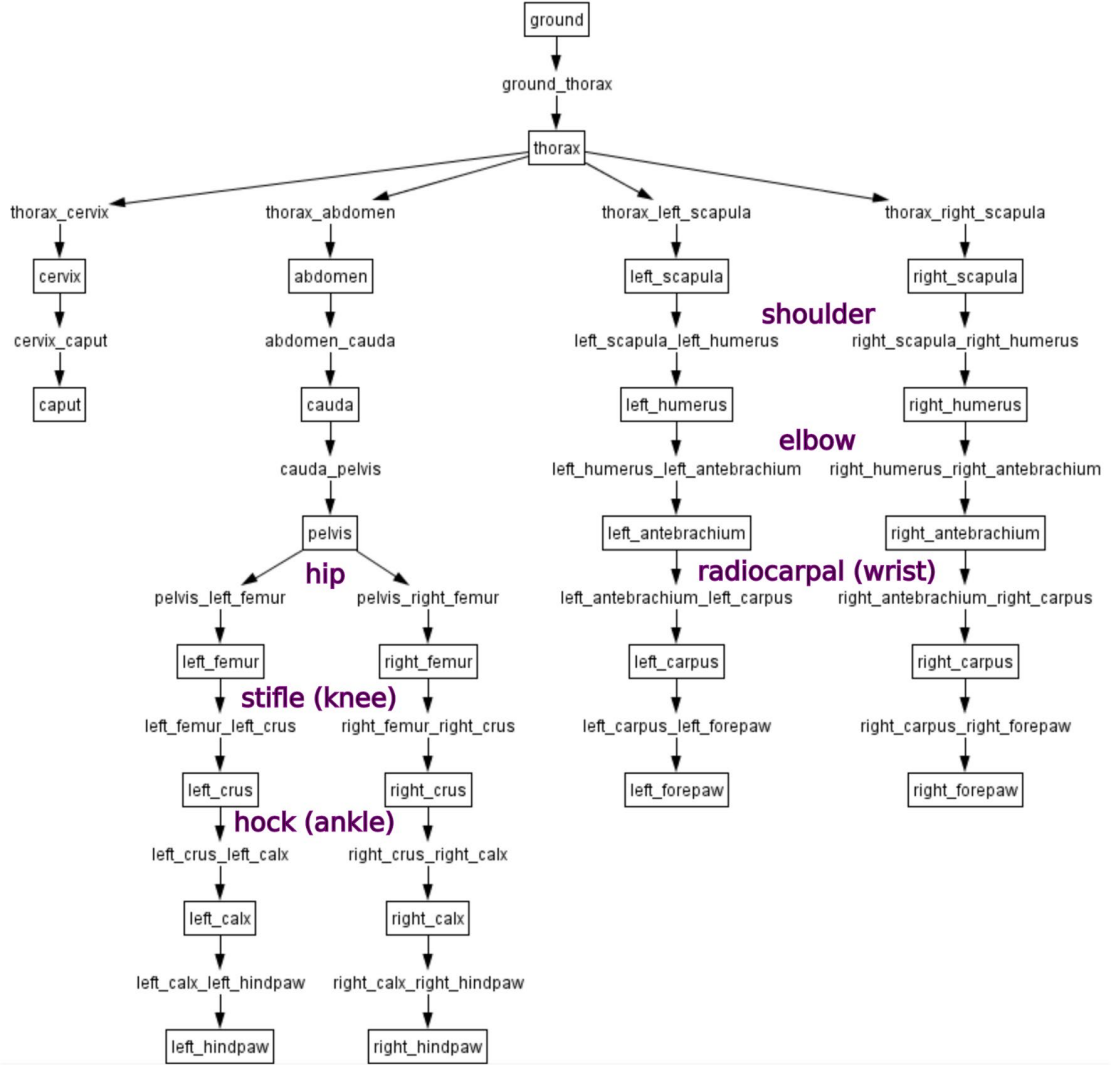
Topology of the segments (boxes) and joints (arrows) of the whole Beagle (BE) model using the software package OpenSim^28,29^.

In the scripts, the segments are arranged hierarchically to build a kinematic chain as displayed in Fig. 6. The most proximal segment is joined to the ground (the thorax in the case of the whole). The scripts include additional data for the relative position and orientation of the segments (bones), the segment masses, the centre of mass, and the inertia (bones-model). The individual sub-models (fore- or hindlimbs) contain the mass of all segments (skeleton is complete) but only the muscles of their corresponding segments.

**Figure 7:**
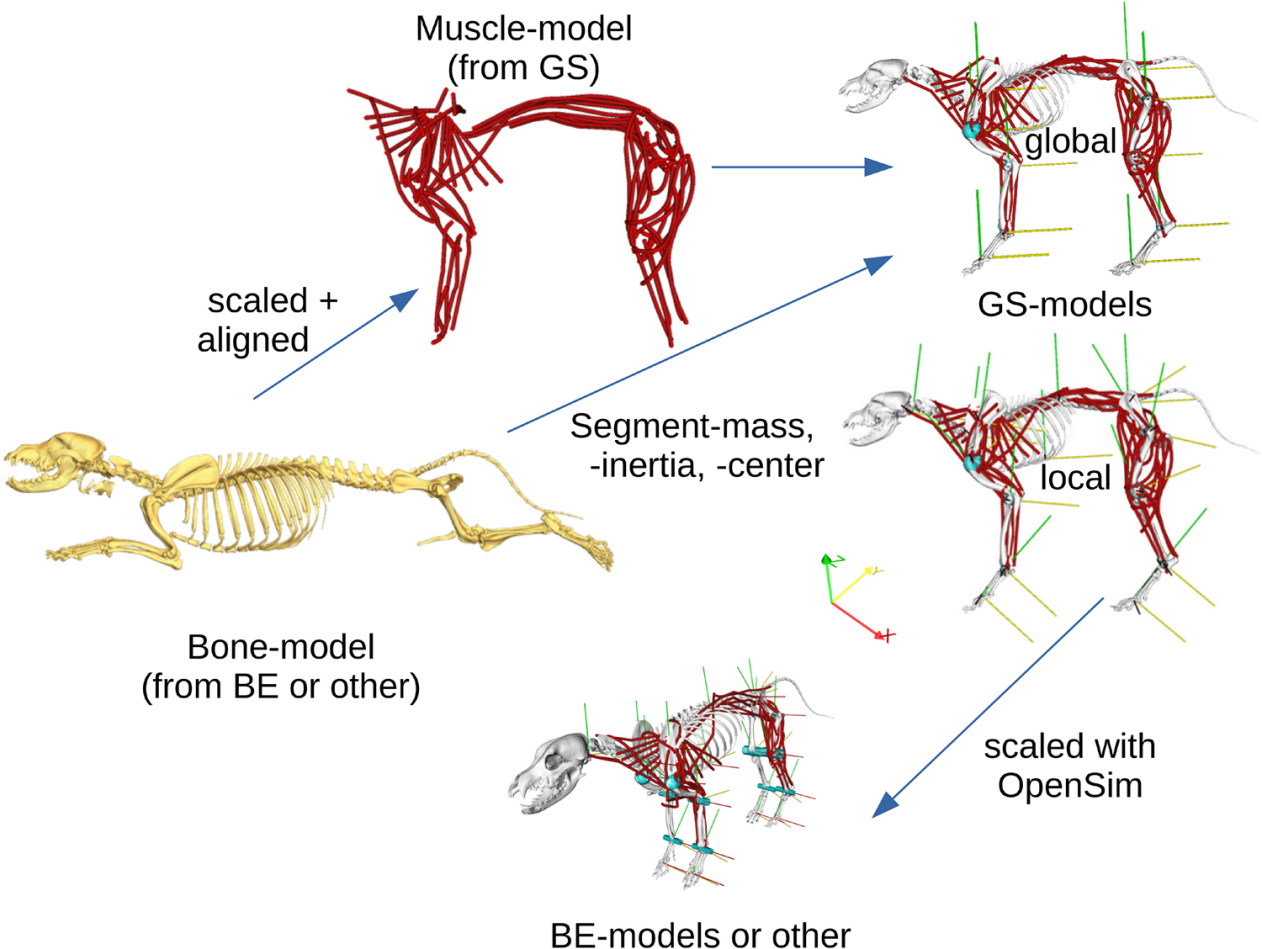
Representation of the model assembly, from the bone model (yellow bones) to the muscle model (red paths) to the resulting simulation model, taking into account the transformations performed. The muscles (red) can be generated as paths closer to the real curves or just straight. Rotation around the red axis (x) represents protraction/retraction or flexion/extension, around the yellow axis (y) abduction/adduction, and around the green axis (z) axial rotation. The sub-figures were created using the software packages Amira^57^ and OpenSim^28,29^.

The bones-model (Fig. 7) is scaled and oriented to fit the muscle model (based on the GS-model). This task was performed in Blender^61^. We scaled the bones model to fit the muscle model because this is easier to do than scaling muscles to fit the bones. In dogs the segment length as a percentage of leg length is approximately the same among different dog breeds including chondrodystrophic dwarf breeds^47,62,63^. Thus, just one size factor is necessary to scale a leg (in our case forelimbs and hindlimbs were multiplied by 1.66). For the spine, neck, and head we obtained a scaling factor of 1.25 (note that the scaling factor among breeds is not available in the literature). After scaling Beagle bones to fit the GS-model, muscle origins and insertions of the muscle model (see Fig. 7) were easily corrected to match those of the Beagle bones’ model (Suppl. 6 SFig. 5 displays the anatomical differences between the scaled Beagle skeleton and that of the German Shepherd). In the basic Master script, scaled BE-model and muscle-line models were combined. The position of the joints, position relative to the joint centre, and joint types were derived from the CT-based BE-model. Additionally, geometrical constraints have been added to the joints to prevent bone penetration by the muscles. Cylinders were used to constrain hinge joints while spheres were used to constrain ball-and-socket joints (Suppl. 6 SFig. 6). Our script permits us to generate curved or straight muscle paths that replicate realistic lines of action. Muscle insertion points are assigned to their corresponding segments. Model segments (bones and muscle insertion points) are then compiled in SIMM in either global or local coordinates. We used here a local coordinate system for every segment.

### Experimental data, kinematics, GRF, and joint torques

3D-kinematic (from motion capture/passive markers) and kinetic data from a previous dog study^30^ were used to develop the inverse simulation solutions. Experimental methods to collect kinematic and kinetic data can be found in that paper. Motion capture data belongs to the same individual from which we took the CT scan. Eight walking strides (joint angles and GRFs from left forelimb) were used as the basis for the analysis. We first computed 3D-Kinematic (XYZ Cardan-sequence) motion from marker data relative to the lab-frame. We then transformed these segment kinematics to joint kinematics by using quaternions because OpenSim^28,29^ necessitates 3D-relative joint coordinates. For this, we first transformed 3D-segmental Cardan angles to quaternions (for formulas see Henderson^64^). Then, we computed the quaternions between adjacent segments by conjugating the quaternion of the lower segment to a joint (i) and multiplying the result by the quaternion of the upper segment to the same joint (i). Afterward, we transformed the results to relative Cardan angles XYZ.

For comparison, joint torques were also computed using the 3D-Newton–Euler method^7,65^, as already presented in Andrada et al.^30^. Here, results are presented in the joint's coordinate systems to allow comparison with OpenSim^28,29^ (see supplementary document for more information, Suppl. 4).

### Joint actuators

Non-biological actuators were placed in all joints to ensure that the static optimization converges. This is important for two reasons. First, joint actuators ‘absorb’ numerical and mass errors. The second was to prevent muscles from exceeding their maximal force. By scanning the parameter space, the optimal joint actuator configuration was determined. As the torque output of the actuators should be as small as possible, we started our scanning at a value 1E−9 (N or Nm). These values were exponentially incremented 1E+1 until a solution was found. After that, actuator values were logarithmically decreased, until the following two conditions were met: (a) the simulation converged to solutions, and (b) all muscles spanning the joints stayed below their maximum forces. Actuator values can be found in Table 1.

### Simulation

To evaluate the model, we estimated inverse dynamics and muscle activation patterns for the forelimbs. Forelimbs are challenging to model due to the high mobility of the scapular joint. Whereas the hindlimbs are linked to the pelvis via a locally static ball-and-socket joint, the scapula is not anchored to the body as a defined joint but via a complex arrangement of extrinsic appendicular muscles. Rather than rotating about a fixed point, scapular motion incorporates both translation and rotation around an instantaneous centre of rotation.

We used OpenSim’s ‘inverse dynamics’ tool^28,29^ to compute the torques in the joints from the kinematic and GRF data. With the ‘static optimization’ tool, we estimated muscle activation patterns and forces. We minimized the sum of muscle activation squared (default configuration) and used the standard cut-off filter configuration (6 Hz) for the kinematic and GRF data. We then compared the torque and muscle activation results with data from the literature. Our goal was to reproduce muscular activation patterns of dog walking with the minimal possible DOFs ‘on’ in every forelimb joint. We started with a sagittal model (every joint represented as a hinge-joint). We analyzed the simulated muscle activations and compared them to the results in the literature. We then increased one DOF in one joint on one plane from the most distal to the most proximal one and mapped again the optimal set of muscle actuators. This procedure was repeated in every joint until the addition of a joint-DOF did not improve simulation results.

### Hierarchical cluster-analysis

In order to evaluate synergistic muscle groups, the predicted muscle activations were further analyzed using the hierarchical cluster analysis^66–68^. This method is typically used to find and group similar patterns within a data set^69^. We first determined the Euclidean distance between the time-series datasets of logarithmic (log2) muscle activations. The Euclidean distance matrix over time was then used by the Ward2 algorithm to analyze dissimilarity in the data and group them. Afterward, a tree was generated based on the minimum distance. The distance was displayed in the dendrogram as length. We used the longest lengths from the root as criteria to sort muscles into groups. Three groups of data had the same length. Subgroups within each group were formed based on their longest length. To perform this analysis we used the software package R^70^ (packages: dendextend, ggdendro and dendsort).

### Data availability

The data that support the findings of this study are available from the authors on reasonable request. The OpenSim model can be downloaded https://simtk.org/projects/dogmodel.

## Supplementary information

Supplementary information.
