# Supplementary material for: A three-dimensional musculoskeletal model of the dog
Source: Sci Rep. 2021 May 31;11:11335. doi: 10.1038/s41598-021-90058-0 (PMC8166944; doi:10.1038/s41598-021-90058-0)
Supplement: Supplementary file 1 — Supplementary information. [file 41598_2021_90058_MOESM1_ESM.pdf]

Supplementary information for: A three-dimensional musculoskeletal model of the dog

Heiko Stark[1,*], Martin S. Fischer[1], Alexander Hunt[2], Fletcher Young[3], Roger Quinn[3], and Emanuel Andrada[1]

[1]Institute of Zoology and Evolutionary Research with Phyletic Museum, Friedrich-Schiller-University Jena, Germany
[2]Department of Mechanical and Material Engineering, Portland State University, United States
[3]Department of Mechanical and Aerospace Engineering, Case Western Reserve University, United States
[*]heiko@starkrats.de

ABSTRACT

The domestic dog is interesting to investigate because of the wide range of body size, body mass, and physique in the many breeds. In the last several years, the number of clinical and biomechanical studies on dog locomotion has increased. However, the relationship between body structure and joint load during locomotion, as well as between joint load and degenerative diseases of the locomotor system (e.g. dysplasia), are not sufficiently understood. Collecting this data through in vivo measurements/records of joint forces and loads on deep/small muscles is complex, invasive, and sometimes unethical. The use of detailed musculoskeletal models may help fill the knowledge gap. We describe here the methods we used to create a detailed musculoskeletal model with 84 degrees of freedom and 134 muscles. Our model has three key-features: three-dimensionality, scalability, and modularity. We tested the validity of the model by identifying forelimb muscle synergies of a walking Beagle. We used inverse dynamics and static optimization to estimate muscle activations based on experimental data. We identified three muscle synergy groups by using hierarchical clustering. The activation patterns predicted from the model exhibit good agreement with experimental data for most of the forelimb muscles. We expect that our model will speed up the analysis of how body size, physique, agility, and disease influence neuronal control and joint loading in dog locomotion.

Contents

1 Import our model in OpenSim 2
2 Mass 3
3 Inertia 3
4 Inverse dynamics (3D-Newton Euler Method) 3
5 Tables 5
6 Figures 6

1 Import our model in OpenSim

To import our model in OpenSim 4.0 or higher

- you need to increase first the heap memory of Java in the OpenSim.conf file
- this file can be generally found under: `C:\OpenSim4.X\etc\OpenSim.conf`
- this file can be open with a text editor, in the code part: `--brandingopensim-J-Xms24m-J-Xmx1024m`
- change 1024 to 2048 or higher depending on your RAM memory `-Xmx1024m->-Xmx2048m` (or higher)
- run OpenSim
- load the low-resolution model: `stark(2021)-beagle-fore_low.osim`
- Done!

To import our model in OpenSim 3.3

- just load `stark(2021)-beagle-fore.osim` (high resolution model)

2 Mass

The volume data from Amit et al (2009) and computed tomography (CT) data from Andrada (unpub. data) were used to calculate the mass of the segments. The individual segments were calculated as percentages of the total body weight of the beagle Simon (13.8 kg).

3 Inertia

For the calculation of the moment of inertia, the function "calculate.inertia.points" of the free program cloud2 was used. This function determines the symmetric inertia matrix for the individual segments on the basis of the point clouds of the bone geometries and the segment masses. A complete segmentation of a calibrated CT data set was not available.

A) Moment of Inertia about CoM 10-3 [kg m²]		
0.435		
	0.329	
		0.254

B) Moment of Inertia about CoM 10-3 [kg m²]		
0.513	0.005	-0.003
	0.500	0.031
		0.044

C) Moment of Inertia about CoM 10-3 [kg m²]		
0.332	0.001	0.001
	0.256	0.035
		0.178

Table 1. Moment of inertia of the humerus segment of a beagle based on A) Andrada et al. 2017 B) Bone geometries C) Segmentation of computed tomography (CT) data.

The influence of asymmetrical mass distribution was tested using the humerus. The humerus with the surrounding musculature was segmented and the moment of inertia was calculated with the function "scalar.inertia" of the free software imagexd. The comparison showed that the component I_{zz} of the calculation from the bone geometries showed the greatest difference (STab. 1). This can be explained by the fact that the real geometries along the x and y axis were neglected. However, the main moment is determined by the longitudinal direction (bone length). The change between tensors in simulations did not significantly influence either joint-torque nor muscle activation profiles.

4 Inverse dynamics (3D-Newton Euler Method)

This method is based on Winter 2010, and has been presented in Andrada et al., 2017. Synchronized 3-D kinematics, kinetics, and segmental properties were processed and combined in a customized Matlab program. The 3-D coordinates of marker trajectories were smoothed using a fourth-order Butterworth low-pass filter with a cutoff frequency of 6 Hz. To obtain 3-D angular kinematics, a Cardan sequence of 3 rotations around the x, y, and z axes was used as described. The transformation matrix for the Cardan sequence was as follows:

$$\begin{pmatrix} x_3 \\ y_3 \\ z_3 \end{pmatrix} = \begin{pmatrix} c_2c_3 & s_3c_1 + s_1s_2c_3 & s_1s_3 - c_1s_2c_3 \\ -c_2c_3 & c_1c_3 - s_1s_2s_3 & s_1c_3 + c_1s_2s_3 \\ s_2 & -s_1c_2 & c_1c_2 \end{pmatrix} \begin{pmatrix} x_0 \\ y_0 \\ z_0 \end{pmatrix} \tag{1}$$

where the indices *1,2,3* indicate the angles $\theta_1, \theta_2, \theta_3$. For example c_1 means $cos(\theta_1)$. The angles $\theta_1, \theta_2, \theta_3$ can thus be obtained from the trigonometric relations present in the transformation matrix. Those angles and their time derivatives are expressed in global coordinates. For the purpose of this study, we were interested in segmental (body-frame) kinematics and dynamics. To transform velocities expressed in laboratory frame into body frame, the following transformation matrix was used:

$$\begin{pmatrix} \omega_x \\ \omega_y \\ \omega_z \end{pmatrix} = \begin{pmatrix} c_2c_3 & s_3 & 0 \\ -c_2c_3 & c_3 & 0 \\ s_2 & 0 & 1 \end{pmatrix} \begin{pmatrix} \dot{\theta}_1 \\ \dot{\theta}_2 \\ \dot{\theta}_3 \end{pmatrix} \tag{2}$$

where the angular accelerations in body frame (α_x, α_y, and α_z) are simply the time derivatives of the respective velocities (θ_x, θ_y, and θ_z). Then, the force (resp. torque) around the proximal end of each segment was estimated by use of the Newton-Euler equations of motion in the centre of motion (CoM) as follows:

$$R_{xp} = R_{xd} + m\ddot{x} \tag{3}$$

$$R_{yp} = R_{yd} + m\ddot{y} \quad (4)$$

$$R_{zp} = R_{zd} + m\ddot{z} + mg \quad (5)$$

$$M_{xp} = I_1\alpha_x + (I_3 - I_2)\omega_z\omega_y + R_{yd}l_d + R_{yp}l_p + M_{xd} \quad (6)$$

$$M_{yp} = I_2\alpha_y + (I_1 - I_3)\omega_x\omega_z + R_{xd}l_d + R_{xp}l_p + M_{yd} \quad (7)$$

$$M_{zp} = I_3\alpha_z + (I_2 - I_1)\omega_y\omega_x + M_{zd} \quad (8)$$

where m is the mass of the segment, I is the principal moment of inertia of the segment around the CoM, R is the reaction force, M is the torque in the proximal (p) and distal (d) ends of the segment, and l is the lever arm of the forces relative to the CoM.

5 Tables

Forelimb	Torso	Hindlimb
M. trapezius pars cervicalis	M. longissimus	M. psoas major
M. trapezius pars thoracica	M. quadratus lumborum	M. psoas minor
M. rhomboideus pars thoracica	M. iliocostalis	M. adductor longus
M. rhomboideus pars cervicalis	M. sacrocaudalis	Mm. gemelli
M. latissimus dorsi		M. gluteus medius
M. brachiocephalicus [1 - 2]		M. gluteus profundus
M. serratus [1 - 12]		M. gluteus superficialis
M. pectoralis profundus		M. iliacus
M. pectoralis superficialis		M. obturator externus
M. supraspinatus		M. obturator internus
M. infraspinatus		M. piriformis
M. subscapularis		M. pectineus
M. deltoideus pars scapularis		M. sartorius caudalis
M. deltoideus pars acromialis		M. sartorius cranialis
M. teres major		M. tensor fasciae latae
M. teres minor		M. quadratus femoris
M. coracobrachialis		M. vastus lateralis
M. biceps brachii		M. vastus medialis
M. brachialis		M. rectus femoris
M. triceps brachii caput longum		M. abductor cruris
M. triceps brachii caput laterale		Mm. adductores
M. triceps brachii caput accessorium		M. biceps femoris caput longum
M. triceps brachii caput mediale		M. biceps femoris caput breve
M. anconeus		M. gracilis tractus calcaneus
M. pronator teres		M. semimembranosus
M. supinator		M. semitendinosus
M. flexor carpi radialis		M. gastrocnemius lateralis
M. flexor carpi ulnaris		M. gastrocnemius medialis
M. extensor carpi ulnaris		M. peroneus longus
M. extensor carpi radialis		M. tibialis caudalis
M. abductor pollicis longum		M. tibialis cranialis
		M. popliteus

Table 2. Complete list of all muscles for the whole model and for the sub-models (fore-, hindlimb and torso). The number of sub muscles is shown in brackets.

6 Figures

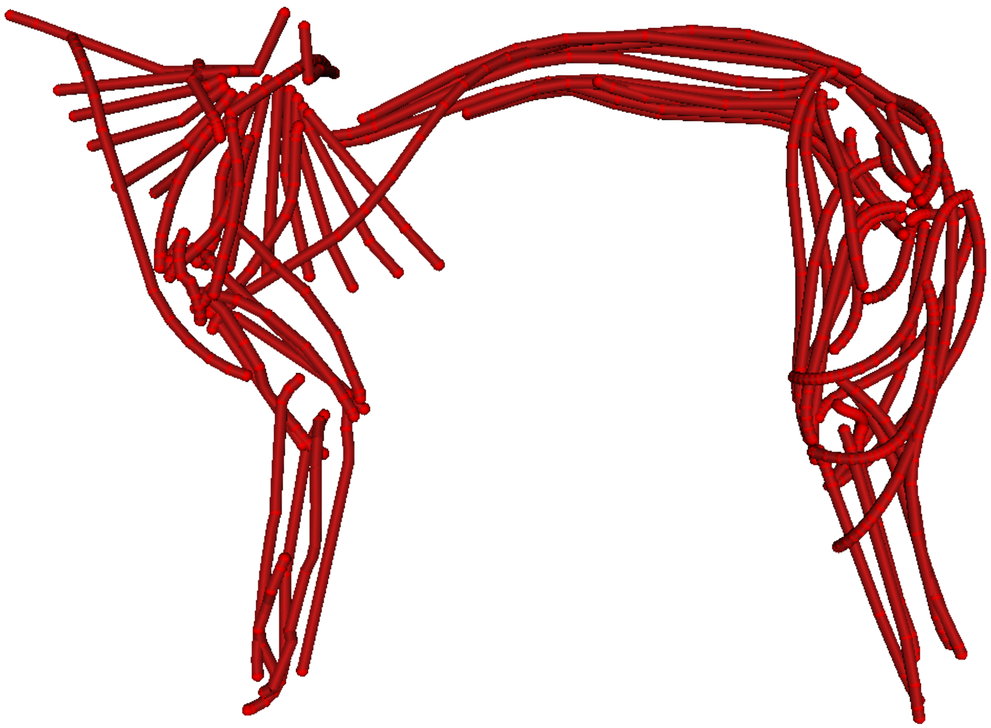

Figure 1. Muscle reconstruction (lateral view) based on the German Shepherd dog (GS) model for the fore and hind limbs as well as some trunk muscles. The figure was created with the software package OpenSim.

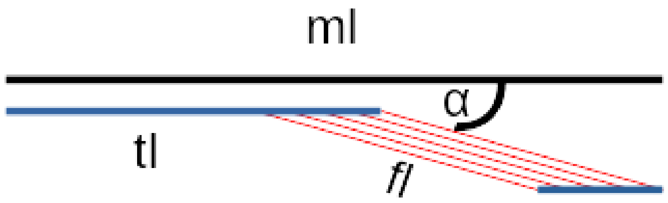

Figure 2. Illustration of the muscle parameter set for the tendon length calculation. The figure was created with the software package LibreOffice.

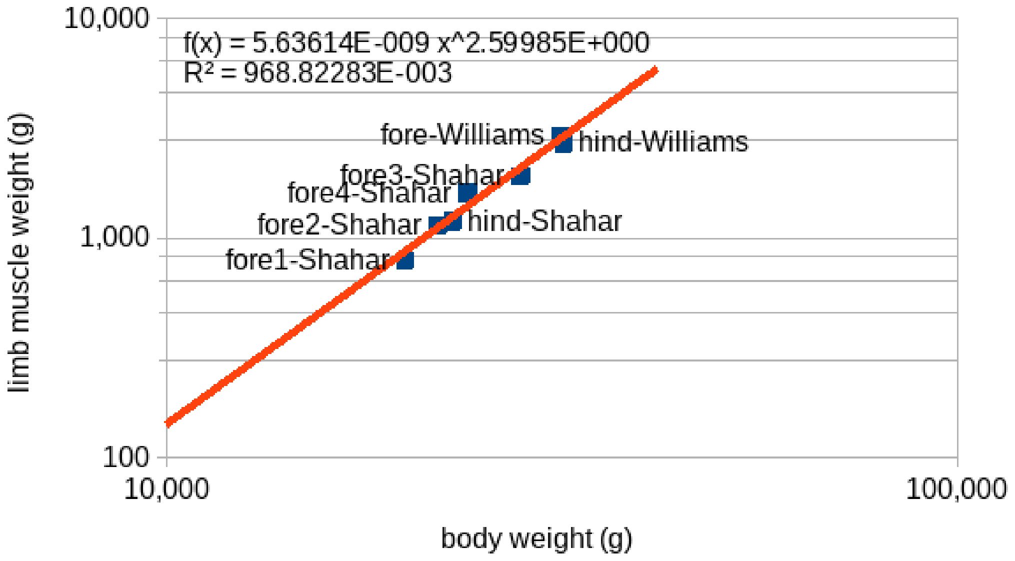

Figure 3. Comparison of the different total muscle masses and body weight between the dogs/studies (log. scale) for Shahar & Milgram (2001 & 2005) and Williams et al. (2008a & 2008b). The figure was created with the software package LibreOffice.

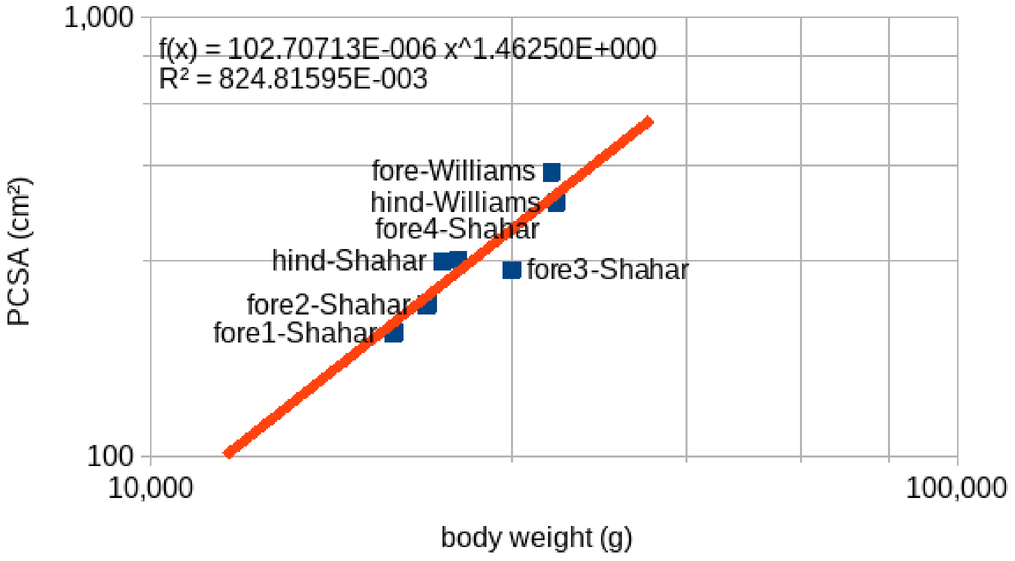

Figure 4. Comparison of the physiological cross-sectional areas (PCSA) and body weight between the dogs/studies (log. scale) for Shahar & Milgram (2001 & 2005) and Williams et al. (2008a & 2008b). This demonstrates that total leg muscle PCSA scales with body mass, and enables us to scale data from different breeds for use in our BE-model. The figure was created with the software package LibreOffice.

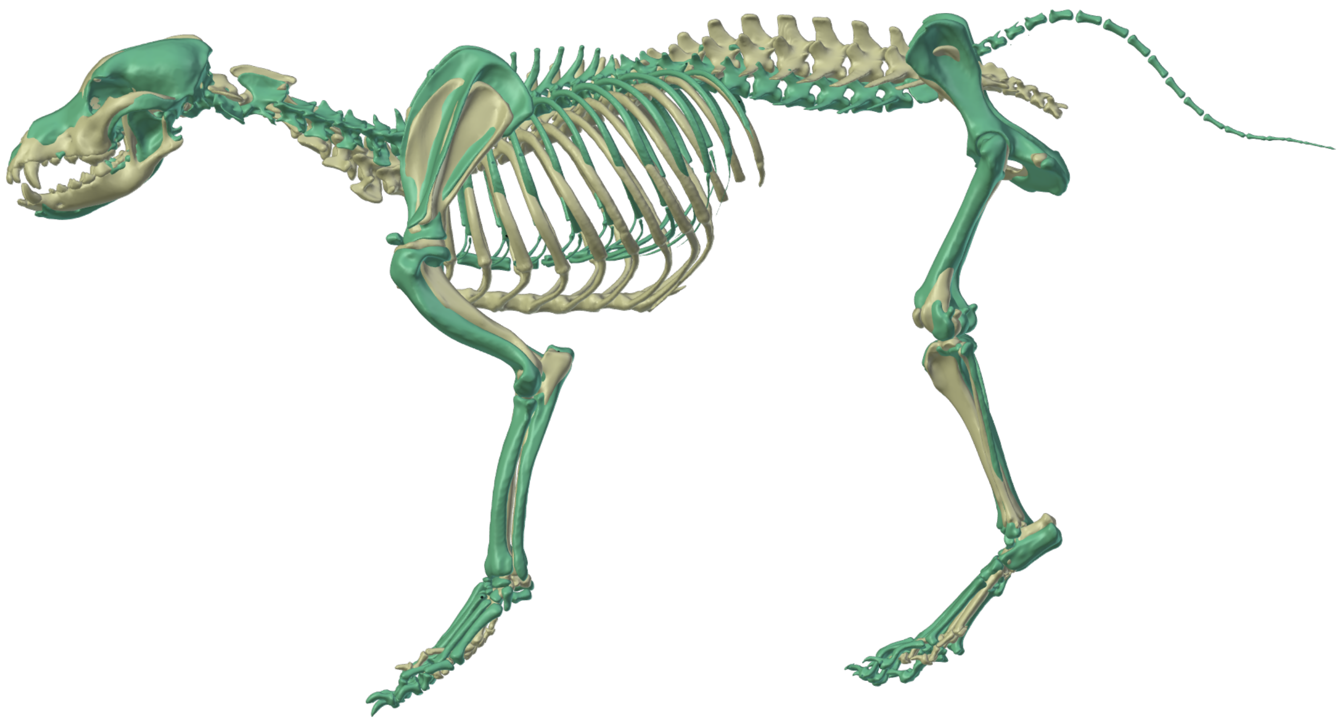

Figure 5. Visualization of the reconstructed bones of the German Shephard (GS) model (grey) and the Beagle (BE) model (green). The BE model was scaled to the size of the GS model. The figure was created with the software package Blender.

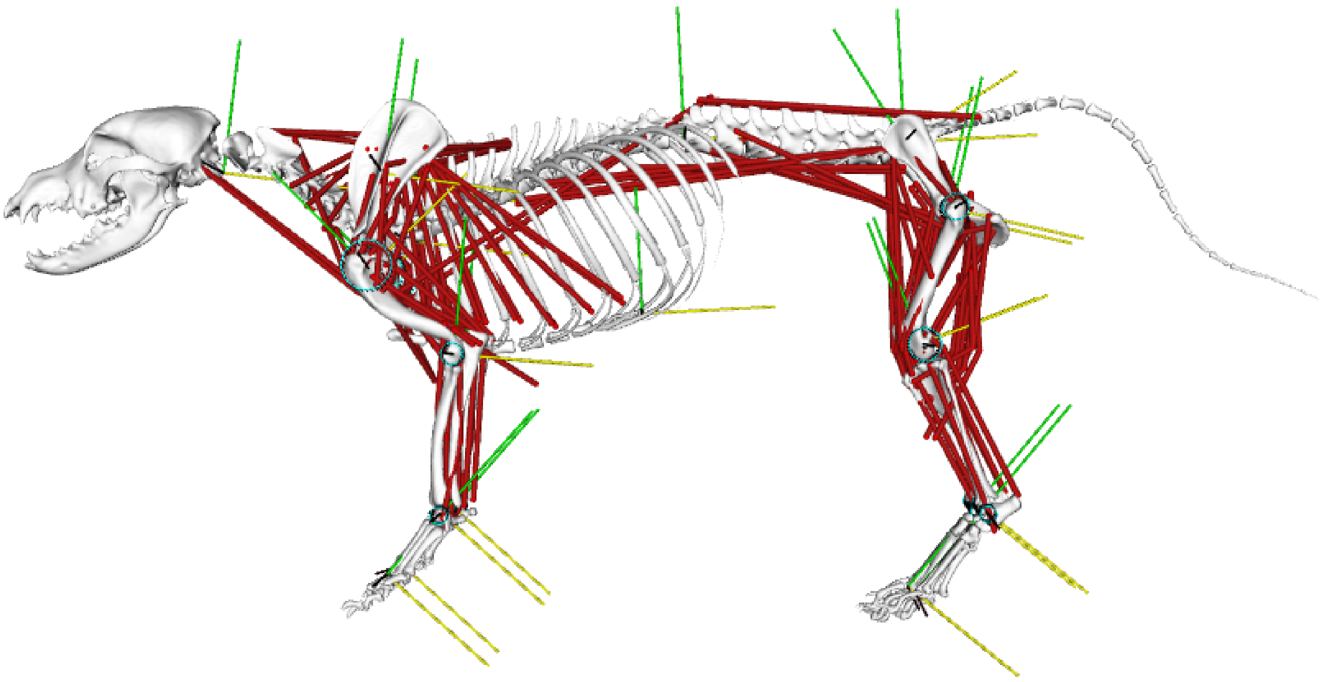

Figure 6. Full simulation model of a Beagle (BE-model with linear muscles – red) with visualization of the local coordinate systems (black, green, yellow) and the constraints (cyan). The figure was created with the software package OpenSim.

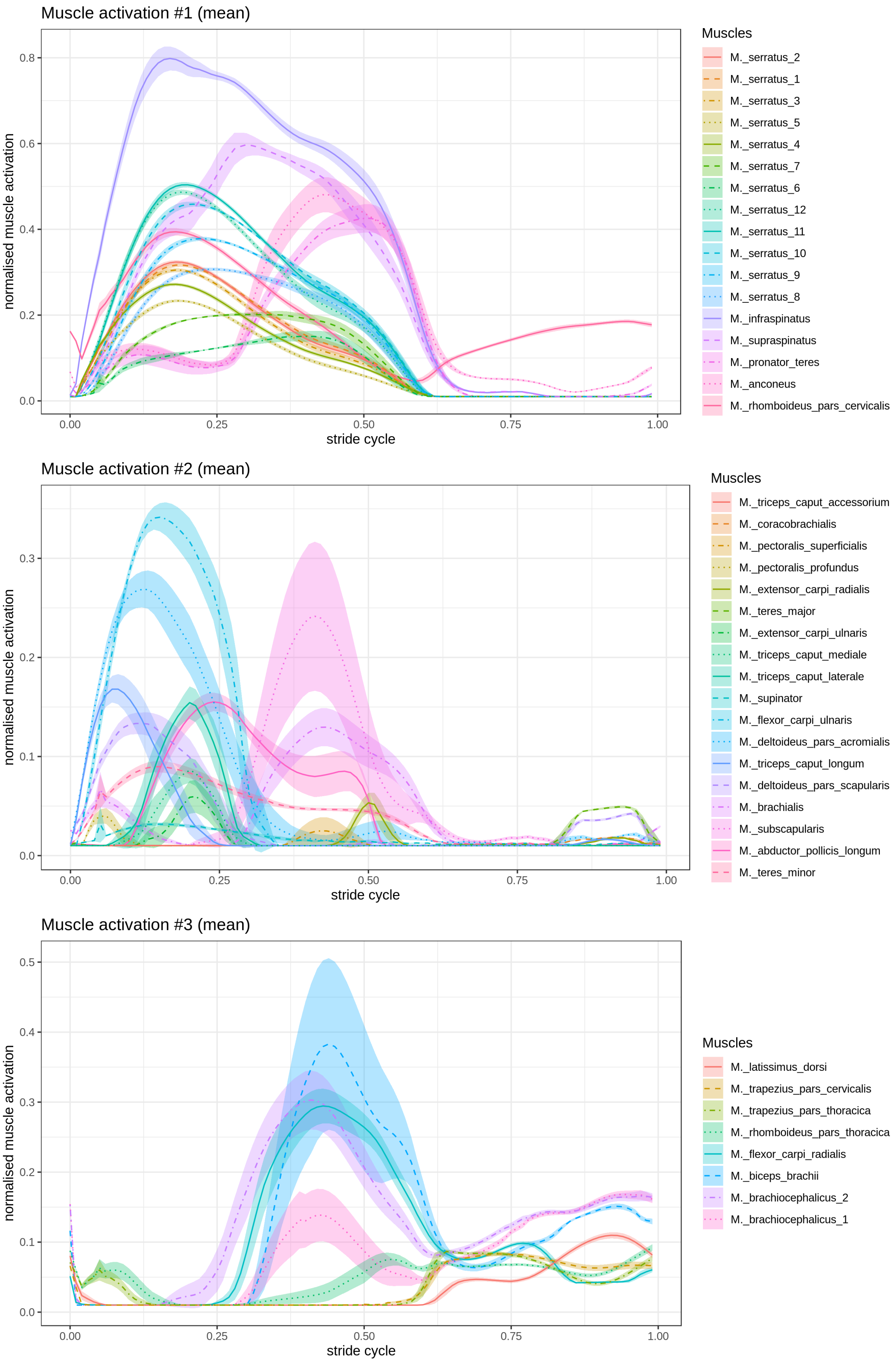

Figure 7. Simulated forelimb muscle activation in a walking Beagle for 43 muscles or muscle parts. The plot shows how individual muscles were activated based on consecutive strides of the same trial. The standard deviation is shown as shaded bands. The muscle groups (#1, #2 & #3) were arranged according to hierarchical clustering (method - ward.d2) and minimal leaf sorting (main text Fig. 7). The figures were created with the software package R.

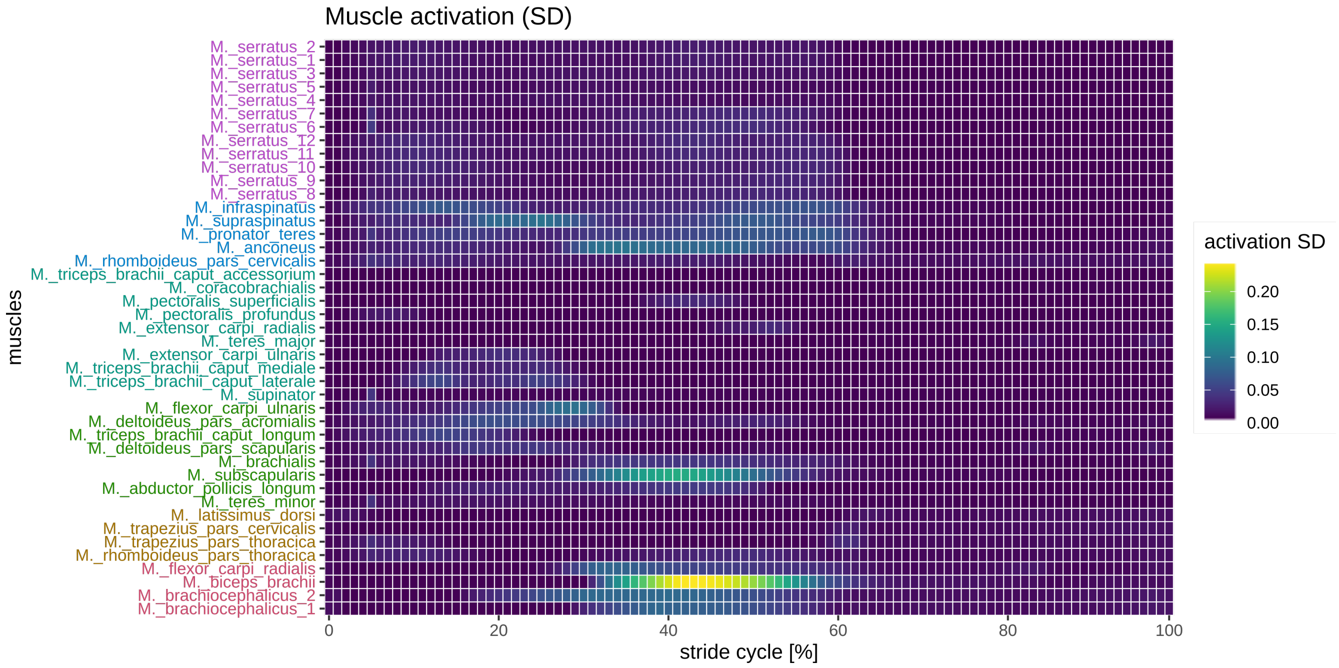

Figure 8. Simulated forelimb muscle activation in a walking Beagle. The plot of the SD values shows how differently individual muscles in simulations were activated based on consecutive strides of the same trial. The lowest SD values are plotted in dark blue, the highest activation values in yellow. The muscle groups (colours, main text Fig. 7) were arranged according to hierarchical clustering (method - ward.d2) and minimal leaf sorting. The figure was created with the software package R.

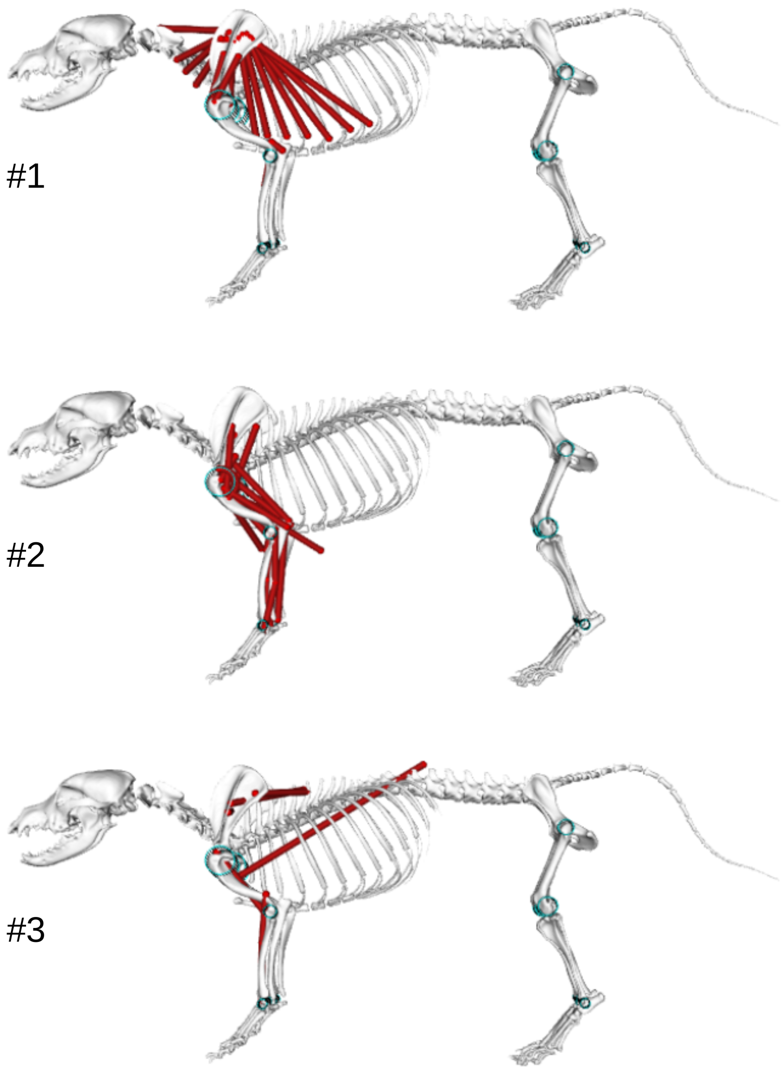

Figure 9. Representation of the muscles according to hierarchical clustering (method – ward.d2) and minimal leaf sorting of simulated forelimb logarithmic muscle activation in the walking beagle (main text Fig. 7). The figures were created with the software package OpenSim.

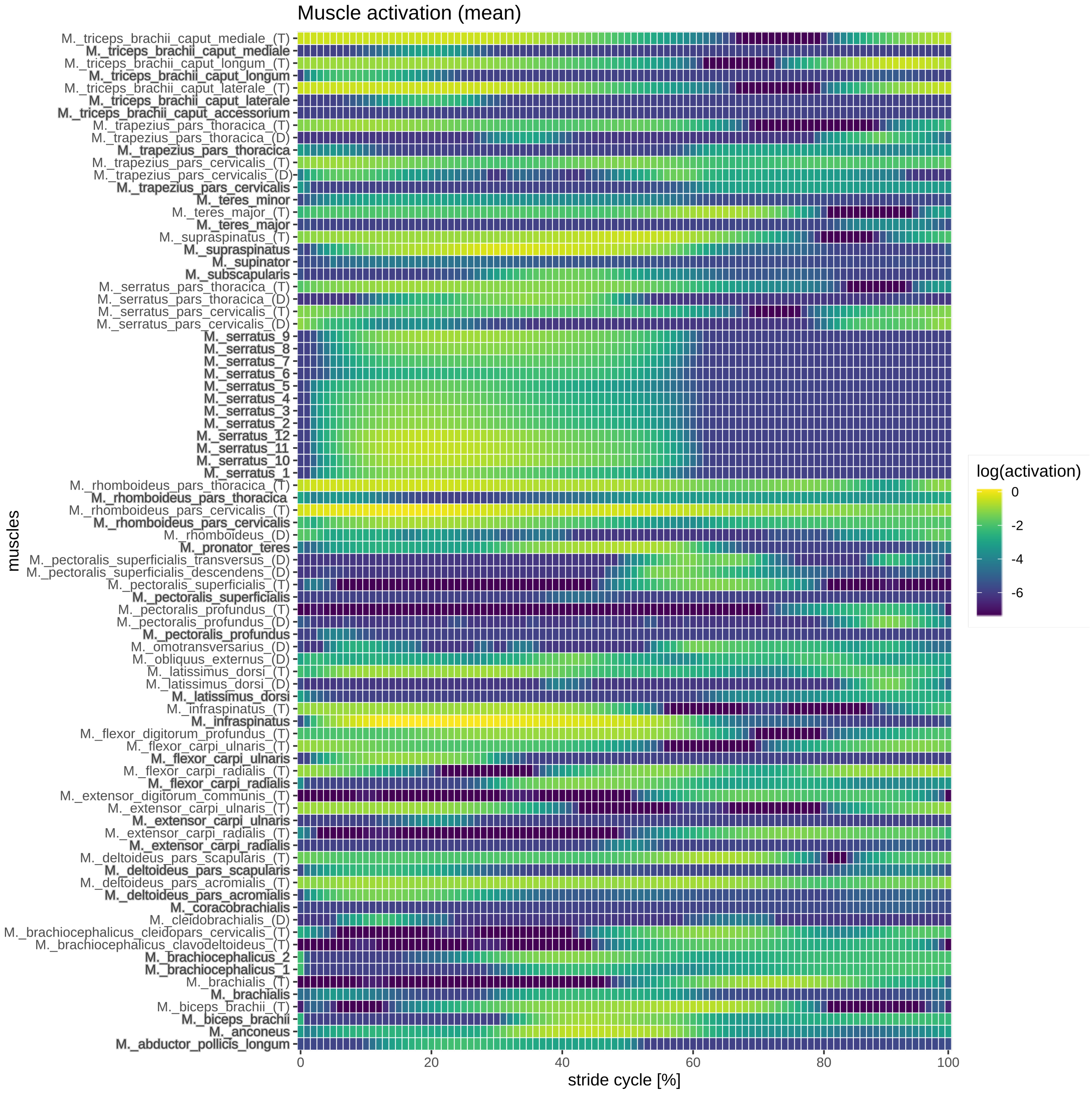

Figure 10. Comparison between literature and simulated forelimb muscle activation (Heat-map). T: Tokuriki, 1973; D: Deban et al., 2012, bold: model. The Lowest activation values are plotted in dark blue, the highest activation values in yellow relative to the stride cycle. Literature data were digitized using WebPlotDigitizer (https://automeris.io/ WebPlotDigitizer/). To permit comparison, the maximal amplitude of the *M. serratus* for all data-sets were normalized to 0.5. Thus, for each data set (T, D, simulation) a different scaling factor was obtained. Every scaling factor was afterwards used to scale all other muscles within each data set. Simulation data are mean values of eight consecutive strides of the same trial. For the plot, data were logarithmic transformed (log2). The figure was created with the software package R.

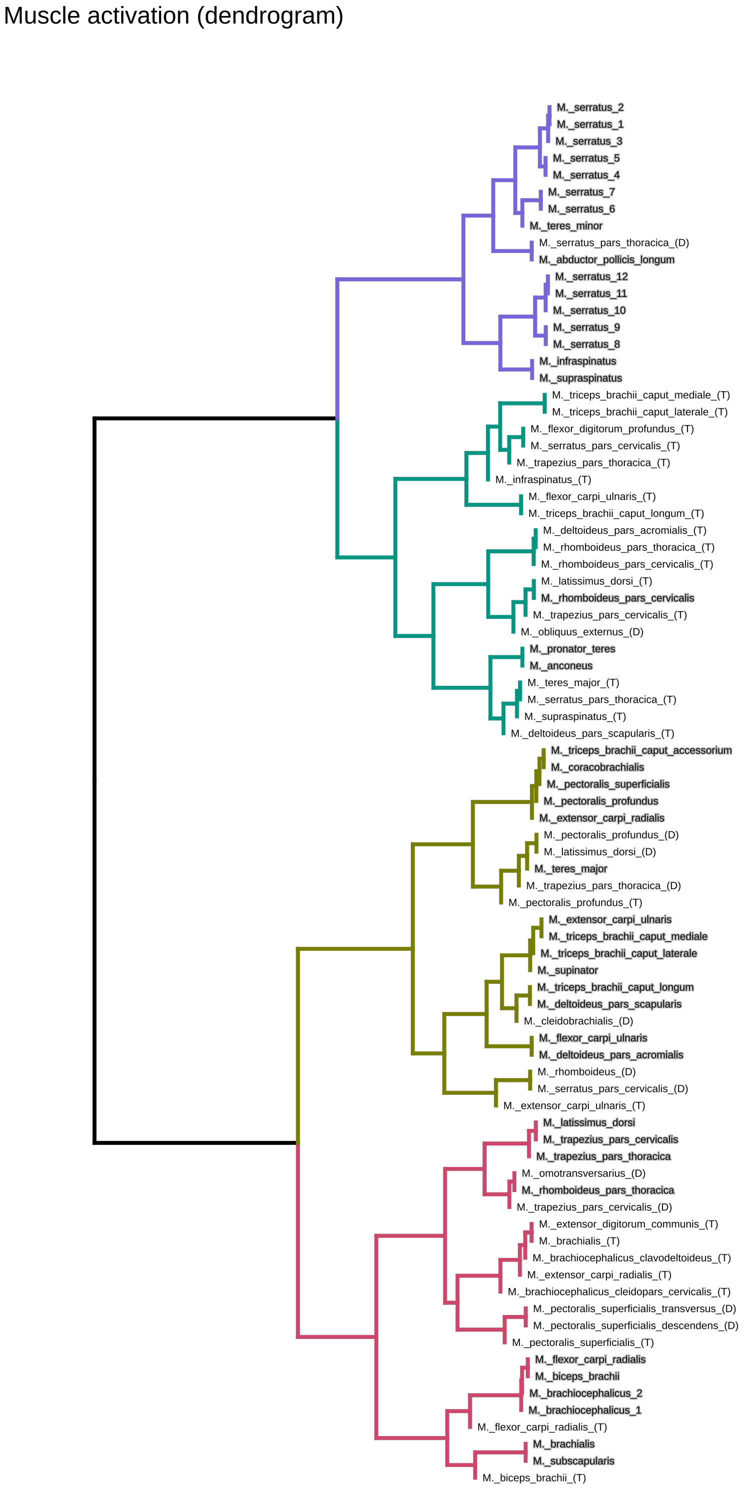

Figure 11. Dendrogram: Comparison between literature and simulated forelimb muscle activation (T: Tokuriki, 1973; D: Deban et al., 2012, bold: model). Literature data were digitized using WebPlotDigitizer (https://automeris.io/WebPlotDigitizer/). To permit comparison, the maximal amplitude of the *M. serratus* for all data-sets were normalized to 0.5. Thus, for each data set (T, D, simulation) a different scaling factor was obtained. Every scaling factor was afterwards used to scale all other muscles within each data set. Simulation data are mean values of eight consecutive strides of the same trial. Activation groups (colours) were arranged according to hierarchical clustering (method - ward.d2) and minimal leaf sorting. The dendrogram represents a distance between different activation patterns. The distance is displayed in the dendrogram as height. We use the largest heights from the root as criteria to separate groups. The figure was created with the software package R.

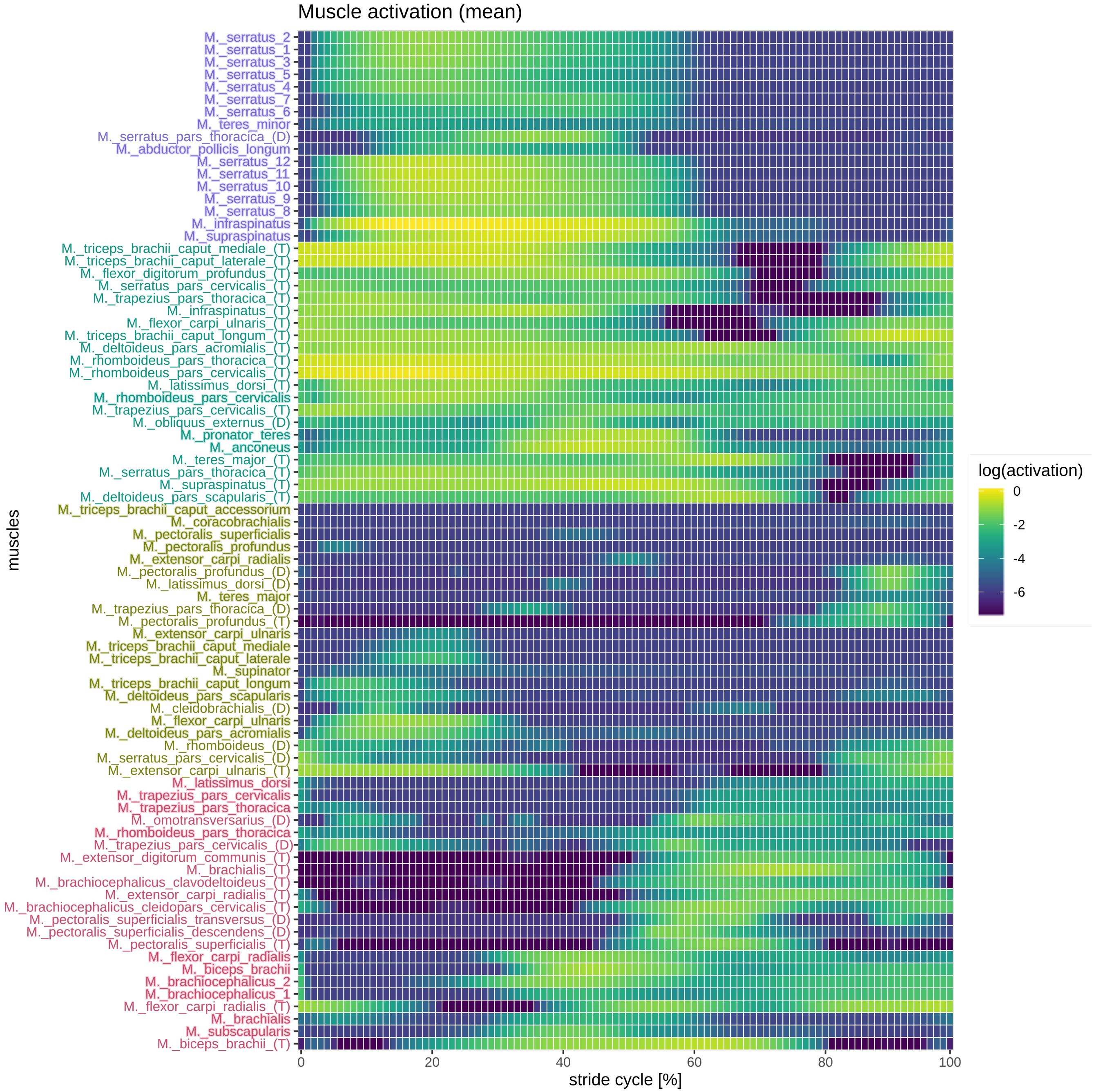

Figure 12. Comparison between literature and simulated forelimb muscle activation (T: Tokuriki, 1973; D: Deban et al., 2012, bold: model) based on hierarchical clustering. Literature data were digitized using WebPlotDigitizer (https://automeris.io/WebPlotDigitizer/). To permit comparison, the maximal amplitude of the *M. serratus* for all data-sets were normalized to 0.5. Thus, for each data set (T, D, simulation) a different scaling factor was obtained. Every scaling factor was afterwards used to scale all other muscles within each data set. Simulation data are mean values of eight consecutive strides of the same trial. Activation groups (colours, see SFig. 11) were arranged according to hierarchical clustering (method - ward.d2) and minimal leaf sorting. For the plot, data were logarithmic transformed (log2). The Lowest activation values are plotted in dark blue, the highest activation values in yellow. The figure was created with the software package R.

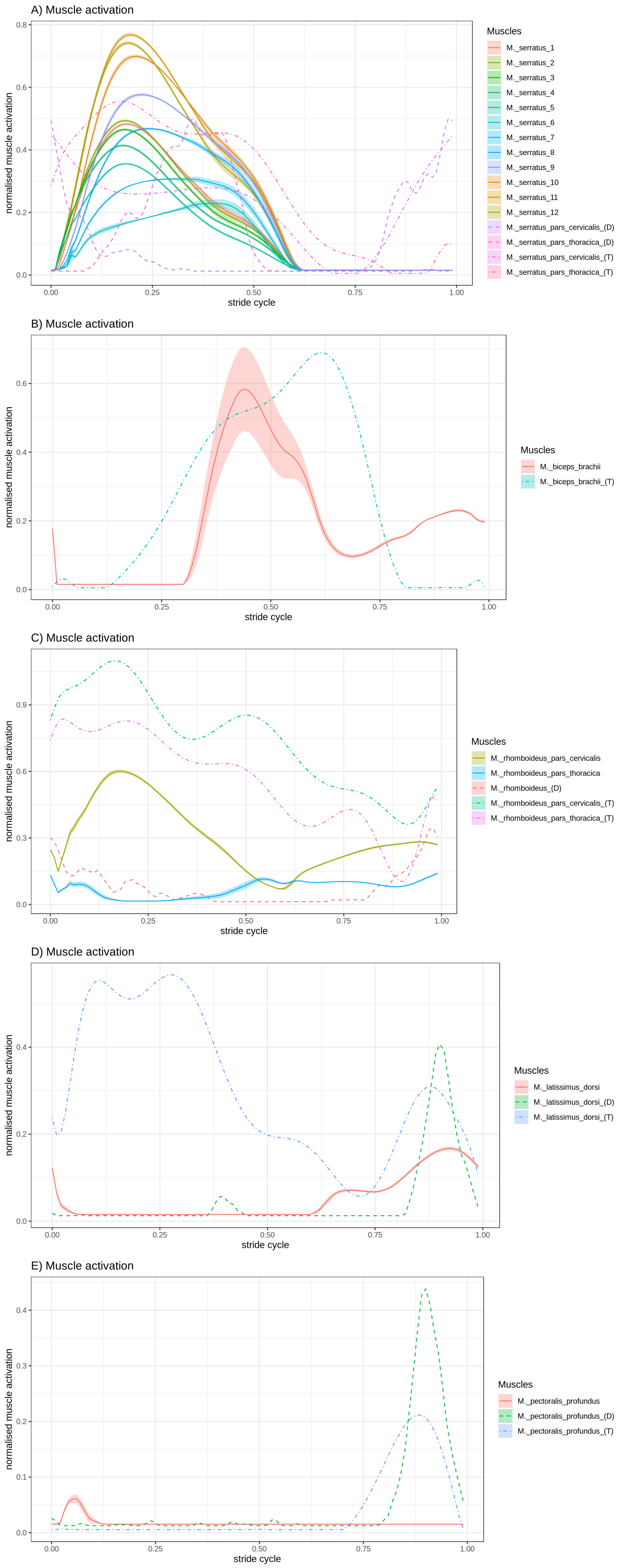

Figure 13. Simulated vs. literature forelimb muscle activation patterns at walk. While *M. serratus* (A), *M. biceps brachii* (B), *M. rhomboideus* (C), and *M. latissimus dorsi* (D) display a relative good match with literature data, *M. pectoralis profundus* (E) does not match. Note also that for the *M. latissimus dorsi* activation patterns reported by Tokuriki (T: 1973) and Deban et al. (D: 2012) differs markedly. Simulation results are mean values of eight consecutive strides. The standard deviation is shown as shaded bands. The figures were created with the software package R.
